# Association between phenotypes and genotypes of antimicrobial resistance in *E. coli* and *Salmonella* isolates from retail chicken meat and live bird market sewage in Bangladesh

**DOI:** 10.1128/spectrum.01062-25

**Published:** 2026-06-30

**Authors:** Mst. Sonia Parvin, Sudipta Talukder, Sayra Tasnin Sharmy, Md. Mehedi Hasan, Md. Taohidul Islam

**Affiliations:** 1 Population Medicine and AMR Laboratory, Department of Medicine, Faculty of Veterinary Science, Bangladesh Agricultural University 54492, Mymensingh, Bangladesh; University of Maryland Eastern Shore, Princess Anne, Maryland, USA; Biological Sciences Department, Delaware State University, Dover, Delaware, USA

**Keywords:** *E. coli*, *Salmonella*, MDR, phenotypic and genotypic associations, retail chicken meat, LBM sewage, Bangladesh

## Abstract

Retail chicken meat contaminated with *Escherichia coli* and *Salmonella* represents a significant global public health concern, particularly in developing countries like Bangladesh. The presence of antimicrobial-resistant (AMR) strains in retail chicken meat poses a serious health threat to consumers. Therefore, a cross-sectional live bird market (LBM) survey was conducted to determine the spectrum of AMR and association among AMR phenotypes, as well as between phenotypes and resistance genes in *E. coli* and *Salmonella* isolated from retail chicken meat (*n* = 320) and LBM sewage (*n* = 64) samples collected from 64 LBMs of 32 upazilas in 16 districts spanning 8 administrative divisions of Bangladesh. *E. coli* and *Salmonella* were identified using culture-based and molecular methods and were subsequently subjected to antimicrobial susceptibility testing. Genes encoding β-lactamases and plasmid-mediated quinolone resistance were detected by multiplex PCR and confirmed by sequencing. Multidrug resistance (MDR) was observed in 99.1% and 92.2% of *E. coli* and in 99.4% and 100% of *Salmonella* from retail chicken meat and LBM sewage samples, respectively. The most frequently detected resistance genes were *bla_TEM*, *bla_CTX-M-1*, *qnrA*, *qnrB*, and *qnrS*. Strong associations between phenotypic resistance and corresponding resistance genes were observed for cefepime*/bla_CTX-M-1*, ceftazidime/*bla_CTX-M-1*, aztreonam**/***bla_CTX-M-1*, cefuroxime*/qnrA,* ceftriaxone*/qnrA,* cefoxitin*/qnrB*, cefixime/*qnrB*, cefaclor/*qnrB*, trimethoprim-sulfamethoxazole/*qnrS*, ampicillin/*qnrS*, and amoxicillin-clavulanic acid*/qnrS* (*P* < 0.05). Overall, these findings indicate that retail chicken meat serves as a potential source of MDR *E. coli* and *Salmonella* harboring clinically relevant AMR genes. Urgent attention is needed, including strengthening antimicrobial stewardship and surveillance programs in poultry production systems in Bangladesh.

**IMPORTANCE:**

Retail chicken meat is a potential reservoir of antimicrobial-resistant (AMR) *Escherichia coli* and *Salmonella*, which pose a major public health concern due to their ability to harbor a wide range of AMR genes. Given the high consumption of chicken meat and the increasing prevalence and invasiveness of multidrug-resistant *E. coli* and *Salmonella*, continuous monitoring of their prevalence and AMR patterns in retail chicken meat and sewage samples is essential. Understanding the associations among resistance genes may provide critical insights for developing effective antimicrobial stewardship and prudent-use guidelines. Moreover, these findings could serve as a foundation for future research addressing public health and food safety challenges associated with AMR foodborne bacteria.

## INTRODUCTION

Antimicrobial resistance (AMR) poses significant problems to public health, with AMR-associated bacterial infections causing hundreds of thousands of deaths annually worldwide (1). The problem is exacerbated by the extensive exchange of antimicrobial resistance genes (ARGs), collectively known as the resistome, between pathogenic and commensal bacteria across diverse ecological niches (2, 3). Resistant bacteria and their associated ARGs can disseminate through direct contact between humans, animals, and environmental microbiomes. Although many studies have focused on major human pathogens, including *Salmonella*, *Campylobacter* spp., and *Escherichia coli* O157 (4), increasing evidence indicates that commensal bacteria also harbor transmissible genetic elements encoding AMR (3, 5, 6). The transfer of resistance genes from nonpathogenic to pathogenic bacteria within the gastrointestinal tract may play a critical role in the emergence and establishment of AMR (3).

AMR is of particular concern in both zoonotic pathogens and commensal bacteria because ARGs can be mobilized through horizontal gene transfer mediated by mobile genetic elements, including plasmids, transposons, integrons, and gene cassettes (3, 5). These elements facilitate the dissemination of multiple resistance genes and enable gene exchange between pathogenic and nonpathogenic bacteria (6, 7). As a result, selection for resistance to multiple antimicrobial classes can occur simultaneously. Numerous studies have demonstrated that ARGs and AMR bacteria, mainly *E. coli* and *Salmonella*, are widely distributed among humans, animals, and environmental reservoirs (2, 3, 6). These organisms possess diverse and complex mechanisms for acquiring and transmitting genetic determinants of resistance and therefore serve as important reservoirs of transmissible resistance (5, 6). Investigating the genetic determinants of resistance in commensal *E. coli* and *Salmonella* is thus essential for improving our understanding of the development and spread of AMR. Elucidating the transmission pathways of ARGs and resistant bacteria remains challenging, as many resistance determinants are commonly detected in *E. coli* and *Salmonella* isolated from both humans and animals (8). Moreover, AMR phenotypes and genotypes can vary among livestock species due to differences in antimicrobial usage practices. Consequently, characterizing AMR at each stage of poultry production is critical for understanding its epidemiology.

Live bird markets (LBMs), where multiple species of live poultry are gathered for trade, represent a key interface linking humans, live poultry, and the environment in urban settings. Individuals with occupational exposure to poultry, such as workers in LBMs and poultry traders, are at increased risk of acquiring AMR bacteria and ARGs (9). Our recent study demonstrated that wholesale chicken markets in Bangladesh play a vital role in disseminating poultry-associated foodborne AMR bacteria and ARGs, particularly β-lactamase (*bla_TEM*, *bla_SHV*, and *bla_CTX-M*) and plasmid-mediated quinolone resistance (PMQR) (*qnrA* and *qnrS*) genes (10). These resistance determinants are clinically significant because they confer resistance to specific antibiotics, limiting treatment options and raising the risk of therapeutic failure (5). The *bla_TEM* gene encodes a β-lactamase that confers resistance to penicillins and first- and second-generation cephalosporins, while *bla_SHV* is associated with cephalosporin resistance, with some variants functioning as extended-spectrum β-lactamases (ESBLs). The *bla_CTX-M* gene encodes ESBLs capable of hydrolyzing third- and fourth-generation cephalosporins and aztreonam, representing a major clinical threat (5). PMQR genes (*qnrA*, *qnrB*, and *qnrS*) encode proteins that protect DNA gyrase and topoisomerase IV from quinolone inhibition, thereby reducing susceptibility to fluoroquinolones (11). The rapid spread of these ARGs in *E. coli* and *Salmonella*, particularly in retail settings like LBMs, facilitates the persistence and evolution of AMR. Retail chicken meat is a well-recognized source of human exposure to these pathogens, often resulting from poor food hygiene practices or the consumption of undercooked meat.

To date, data from Bangladesh on AMR foodborne *E. coli* and *Salmonella* and their ARGs in retail chicken meat isolated from LBMs remain limited and geographically restricted, typically covering only two to three districts (12–17). Additionally, only two studies have reported the occurrence of AMR *E. coli* and *Salmonella* in urban sewage water in Mymensingh district of Bangladesh (18). To our knowledge, no comprehensive study has yet examined the associations between AMR phenotypes and genotypes in *E. coli* and *Salmonella* isolated from retail chicken meat and LBM sewage covering a wide geographic area in Bangladesh. Understanding the relationships among ARGs and between resistance phenotypes and genotypes is critical for informing effective antimicrobial stewardship and prudent-use guidelines. Therefore, the objectives of this study were to determine the spectrum of AMR and associated β-lactamase- and PMQR-encoding genes in *E. coli* and *Salmonella* isolated from retail chicken meat and LBM sewage collected from 64 LBMs across 32 upazilas in 16 districts spanning all 8 divisions of Bangladesh, and to assess potential associations between resistance phenotypes as well as between phenotypic and genotypic resistance profiles.

## MATERIALS AND METHODS

### Isolation and identification of *E. coli* and *Salmonella*

Between 2018 and 2020, 320 retail chicken meat samples and 64 sewage samples were collected from live bird markets located in 32 upazilas across 16 districts in 8 divisions of Bangladesh. *E. coli* and *Salmonella* were isolated and identified following standard bacteriological procedures (19, 20). Briefly, 25 g of each chicken meat sample was chopped into fine pieces, aseptically transferred into a sterile glass beaker containing 225 mL of buffered peptone water (BPW), homogenized for 2 min, and incubated at 37°C for 18 h. Furthermore, bacteria were pelleted by centrifugation of sewage water samples at 600 × *g* for 20 min, re-suspended in 1 mL of BPW, and incubated at 37°C for 18 h for pre-enrichment of bacteria. Following pre-enrichment, aliquots from each sample were transferred into two separate test tubes: one containing the nutrient broth (NB) for the isolation of *E. coli* and another one containing Rappaport-Vassiliadis Soya broth (RVS) for the isolation of *Salmonella*. These cultures were incubated for 24 h at 37°C for selective enrichment (21). Subsequently, a loopful of NB culture was streaked onto Eosin Methylene Blue agar in duplicate for *E. coli*, while a loopful of RVS culture was streaked onto xylose-lysine-deoxycholate agar in duplicate for *Salmonella*. Plates were then incubated at 37°C for 18–24 h. Presumptive colonies were biochemically confirmed using triple sugar iron agar, catalase, oxidase, indole, methyl red, and Voges-Proskauer tests. Molecular confirmation was performed by simplex polymerase chain reaction (PCR). For *E. coli*, amplification of the *malB* promoter gene (585 bp) was conducted using primers ECO-1 (5′-GACCTCGGTTTAGTTCACAGA-3′) and ECO-2 (5′-CACACGCTGACGCTGACCA-3′) (22). For *Salmonella*, amplification of the *ITS* gene (312 bp) was performed using primers ITSF (5′-TATAGCCCCATCGTGTAGTCAGAAC-3′) and ITSR (5′-TGCGGCTGGATCACCTCCTT-3′) (23). Amplification reactions and PCR conditions were as described previously (10). After amplification, PCR products were analyzed by gel electrophoresis on a 1.5% UltraPure Agarose gel stained with ethidium bromide (5 μg/mL) alongside a 100-bp DNA ladder (BioLabs, New England) and visualized under a UV transilluminator and photographed. All PCR-confirmed *E. coli* and *Salmonella* isolates were preserved in nutrient broth containing 50% (vol/vol) glycerol at –20°C for further study.

### Antimicrobial susceptibility testing

Antimicrobial susceptibility was assessed using the Kirby-Bauer disk diffusion method in accordance with Clinical and Laboratory Standards Institute (CLSI) guidelines (24). A total of 31 antimicrobial agents representing 15 antimicrobial classes were tested (Table S1). For colistin and polymyxin B, minimum inhibitory concentrations were determined using the broth microdilution method. Interpretation of results followed CLSI breakpoints (24); when CLSI breakpoints were unavailable (pefloxacin, cephalexin, cephradine, colistin, and polymyxin B), European Committee on Antimicrobial Susceptibility Testing guidelines were applied (25). *E. coli* ATCC 25922 and *Salmonella* Enteritidis ATCC 13076 were used as quality control strains. MDR was defined as the isolate being resistant to at least one agent in three or more antimicrobial classes.

### Detection of β-lactamase and PMQR genes

Two multiplex PCR assays were employed to detect the presence of seven antimicrobial resistance genes, including β-lactamase genes conferring resistance to broad-spectrum β-lactamases (BSBLs) (*bla_TEM* and *bla_SHV*), extended-spectrum β-lactamases (*bla_CTX-M-1* and *bla_CTX-M-2*), and PMQR genes (*qnrA*, *qnrB*, and *qnrS*). Details of the primers, target genes, and amplicon sizes are provided in Table 1 (26, 27). PCR protocols and thermal cycling conditions were followed as described previously (28). Positive controls consisted of previously characterized reference isolates harboring β-lactamase and PMQR genes (28, 29), while sterile phosphate-buffered saline served as the negative control in each PCR run. Amplified products were electrophoresed on a 1.5% agarose gel and visualized under ultraviolet light.

**TABLE 1:** Primers used for the detection of β-lactamase (BSBLs and ESBLs) and PMQR genes^*a*^

Genes	Primers	Sequence 5′→ 3′	Size (bp)	References
BSBL-encoding genes				
*bla_TEM*	TEM-410F TEM-781R	GGTCGCCGCATACACTATTCTC TTTATCCGCCTCCATCCAGTC	372	(26)
*bla_SHV*	SHV-287F SHV-517R	CCAGCAGGATCTGGTGGACTAC CCGGGAAGCGCCTCAT	231	
ESBL-encoding genes				
*bla_CTX-M-1*	ctxm1-15F ctxm1-02R	GAATTAGAGCGGCAGTCGGG CACAACCCAGGAAGCAGGC	588	
*bla_CTX-M-2*	ctxm2-39F ctxm2-45R	GATGGCGACGCTACCCC CAAGCCGACCTCCCGAAC	107	
PMQR-encoding genes				
*qnrA*	qnrA-F qnrA-R	ATTTCTCACGCCAGGATTTG GATCGGCAAAGGTTAGGTCA	516	(27)
*qnrB*	qnrB-F qnrB-R	GATCGTGAAAGCCAGAAAGG ACGATGCCTGGTAGTTGTCC	469	
*qnrS*	qnrS-F qnrS-R	ACGACATTCGTCAACTGCAA TAAATTGGCACCCTGTAGGC	417	

^*a*^ BSBLs, broad-spectrum β-lactamases; ESBLs, extended-spectrum β-lactamases; and PMQR, plasmid-mediated quinolone resistance.

### Partial nucleotide sequencing

The Sanger sequencing of the PCR products was performed commercially (Macrogen Inc., Seoul, South Korea). Briefly, amplicons were purified using an ExoSAP-IT enzymatic cleanup method (Thermo Fisher Scientific, USA) according to the manufacturer’s instructions. Sequencing was performed using a Big-Dye Terminator v3.1 Cycle Sequencing Kit on an ABI Prism 3730XL automated DNA Sequencer (Applied Biosystems, USA). A total of 91 PCR products were sequenced, including 16 *E. coli* isolates targeting the *malB* promoter gene, 13 *Salmonella* isolates targeting the *ITS* gene, and 62 ARGs (*bla_TEM*, 16; *bla_SHV*, 1; *bla_CTX-M-1*, 7; *bla_CTX-M-2*, 1; *qnrA*, 15; *qnrB*, 6; and *qnrS*, 16).

### Data analysis

#### Genetic data analysis and phylogenetic construction

Nucleotide sequences were edited and annotated using the program BioEdit version 7.2.5 (30). Sequence identities were confirmed through comparison with GenBank entries using the BLAST program (National Center for Biotechnology Information, USA). All sequences were deposited to GenBank, and accession numbers were obtained (*E. coli*, MT834993−MT835008; *Salmonella*, MT835009−MT835021; *bla_TEM*, MT820227−MT820234 and MT820239−MT820246; *bla_SHV*, MT820299; *bla_CTX-M-1*, MT820249−MT820255; *bla_CTX-M-2*, MT822177; *qnrA*, MT820256−MT820267 and MT820270−MT820272; *qnrB*, MT796965, MT808177, MT820226, and MT820295−MT820297; and *qnrS*, MT820273−MT820278, MT820280−MT820288, and MT820303). Reference and homologous sequences were retrieved from GenBank for comparative analyses and phylogenetic reconstruction. Sequences were aligned using the default configuration of the multiple sequence alignment tool “MUSCLE” embedded in MEGA X software (31). Phylogenetic trees were constructed using the neighbor-joining method with 1,000 bootstrap replicates and the “p distance” substitution model, except for PMQR genes, for which a phylogenetic tree was generated using the maximum likelihood methods with “Tamura-Nei” models. Final phylogenetic trees were edited using the iTOL online tool (32).

#### Association between AMR and ARGs

Descriptive analyses were performed, and variables were recoded as needed for statistical analysis. Each isolate was initially categorized based on the presence or absence of resistance phenotypes and corresponding ARGs. Isolates were further classified according to the presence of at least one resistance gene for each antimicrobial class considered. Chi-square and Fisher’s exact tests (wherever appropriate) were performed to determine significant differences in prevalence and MDR percentage between sample types. Associations between resistance phenotypes (outcome), resistance genes, and between phenotypes and their corresponding ARGs were analyzed using binary logistic regression. ARGs detected in fewer than 2% of isolates were excluded to avoid issues related to model power and convergence. An odds ratio (OR) >1 indicated a positive association, while an OR <1 indicated a negative association between genotypes and phenotypes (or between genotypes). Strong associations were defined as OR ≥10.0 with statistical significance at *P* ≤ 0.05. The statistical software package SPSS version 22.0 (IBM, Somers, NY, USA) was used for the analyses.

## RESULTS

### Prevalence of *E. coli* and *Salmonella*

**Fig 1:**
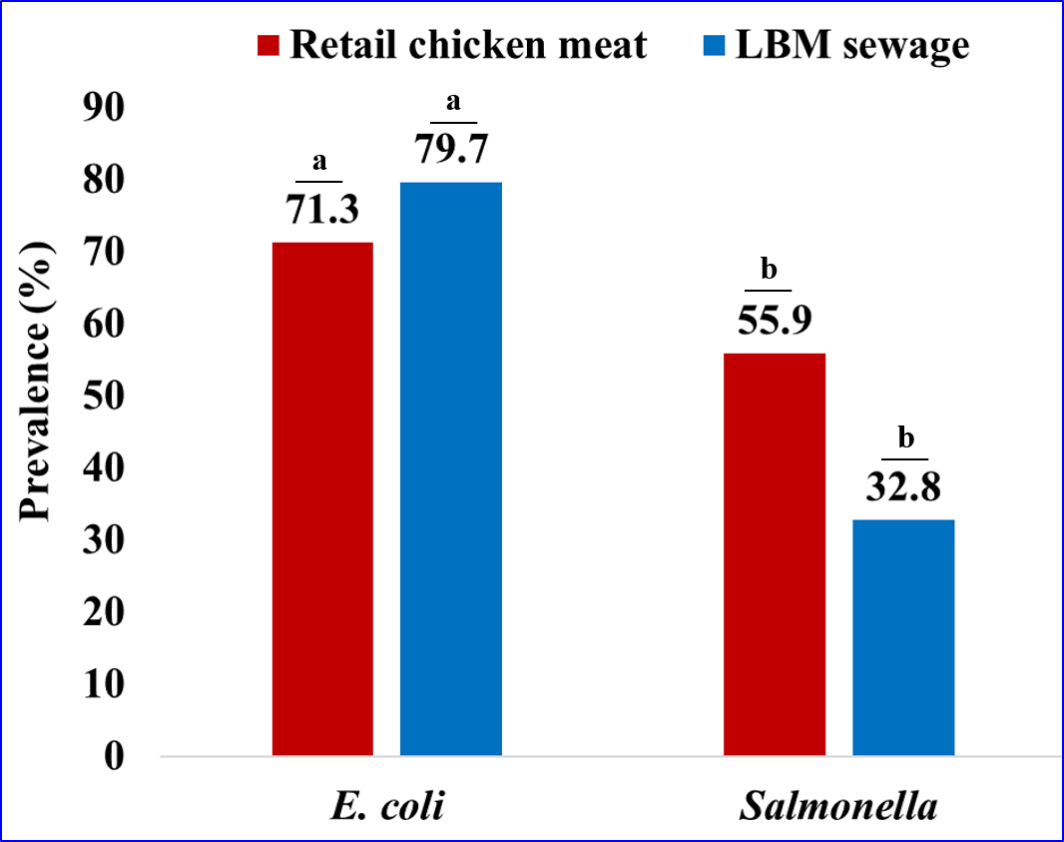
Prevalence of *E. coli* and *Salmonella* isolated from retail chicken meat and live bird market (LBM) sewage samples. Values with different letters differ significantly between sample types (*P* ≤ 0.05).

Among 320 retail chicken meat samples, 228 (71.3%) were positive for *E. coli* and 179 (55.9%) for *Salmonella*. The prevalence of *E. coli* was significantly (*P* ≤ 0.05) higher than that of *Salmonella*. Furthermore, among 64 LBM sewage samples, a substantially higher prevalence of *E. coli* (79.7%, 51/64) was also observed than *Salmonella* (32.8%, 21/64) (Fig. 1). All isolates generated PCR amplicons of the expected fragment size when amplified with species-specific primers.

### Antimicrobial resistance and MDR pattern

**Fig 2:**
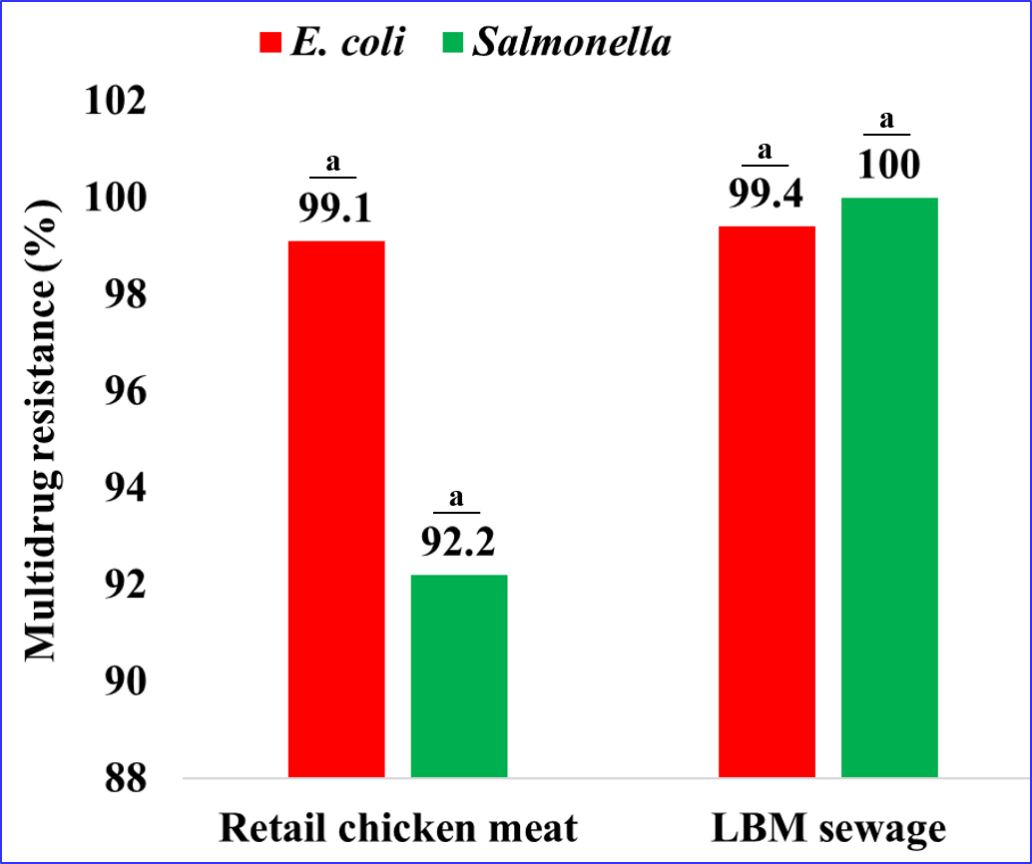
Multidrug resistance patterns observed among *E. coli* and *Salmonella* isolated from retail chicken meat and live bird market (LBM) sewage samples. Values in the same column with the same letter do not differ significantly (*P* > 0.05).

Among *E. coli* isolates, 99.1% (226/228) from retail chicken meat and 92.2% (47/51) from LBM sewage were MDR. Similarly, MDR was observed in 99.4% (178/179) of *Salmonella* isolates from retail chicken meat and in all isolates (100%, 21/21) from LBM sewage (Fig. 2).

Resistance profiles among *E. coli* isolates varied widely, ranging from 0.4% to 98.7% in retail chicken meat and from 2% to 94.1% in LBM sewage. For retail chicken meat (*n* = 228), particularly high resistance was observed to commonly used antimicrobials, including pefloxacin (98.7%), ampicillin (95.6%), trimethoprim-sulfamethoxazole (92.1%), nalidixic acid (79.8%), and doxycycline (74.6%). Fluoroquinolone resistance was also widespread, with 61%–67% of isolates resistant to ciprofloxacin, ofloxacin, gatifloxacin, and levofloxacin (Table 2). In contrast, resistance to tigecycline (0.4%) and cefoxitin (2.6%) was lowest. Notably, substantial resistance to carbapenems was detected with 73.7% and 34.6% of *E. coli* isolates resistant to imipenem and meropenem, respectively. Among isolates from LBM sewage (*n* = 51), the highest resistance was observed for pefloxacin (94.1%), trimethoprim-sulfamethoxazole (86.3%), and ampicillin (82.4%). Resistance to cefoxitin (2%), piperacillin-tazobactam (3.9%), and amikacin (9.8%) remained low, and no isolate was resistant to tigecycline. Alarmingly, carbapenem resistance was extremely high (90.2%), with 58.8% resistant to imipenem and 31.4% to meropenem (Table 2).

**TABLE 2:** Individual antimicrobial resistance pattern of *E. coli* and *Salmonella* isolated from retail chicken meat and live bird market (LBM) sewage samples

Antimicrobial agents	No. of resistant isolates (%)			
	Retail chicken meat		LBM sewage	
	*E. coli* (*n* = 228)	*Salmonella* (*n* = 179)	*E. coli* (*n* = 51)	*Salmonella* (*n* = 21)
Fluoroquinolones				
Ciprofloxacin	153 (67.1)	119 (66.5)	24 (47.1)	7 (33.3)
Nalidixic acid	182 (79.8)	148 (82.7)	30 (58.8)	12 (57.1)
Levofloxacin	139 (61.0)	17 (9.5)	22 (43.1)	1 (4.8)
Norfloxacin	128 (56.1)	63 (35.2)	23 (45.1)	3 (14.3)
Gatifloxacin	150 (65.8)	45 (25.1)	24 (47.1)	3 (14.3)
Pefloxacin	225 (98.7)	163 (91.1)	48 (94.1)	17 (81.0)
Ofloxacin	152 (66.7)	66 (36.9)	28 (54.9)	4 (19.0)
Non-extended spectrum cephalosporins				
1st generation				
Cephalexin	45 (19.7)	37 (20.7)	7 (13.7)	0
Cephradine	45 (19.7)	37 (20.7)	9 (17.6)	2 (9.5)
Cefuroxime	17 (7.5)	27 (15.1)	7 (13.7)	2 (9.5)
2nd generation				
Cefaclor	31 (13.6)	33 (18.4)	8 (15.7)	0
Extended-spectrum cephalosporins				
3rd generation				
Ceftazidime	19 (8.3)	27 (15.1)	4 (7.8)	1 (4.8)
Ceftriaxone	23 (10.1)	22 (12.3)	8 (15.7)	0
Cefotaxime	46 (20.2)	45 (25.1)	21 (41.2)	3 (14.3)
Cefixime	26 (11.4)	29 (16.2)	6 (11.8)	2 (9.5)
4th generation				
Cefepime	17 (7.5)	22 (12.3)	7 (13.7)	0
Cephamycins				
Cefoxitin	6 (2.6)	13 (7.3)	1 (2.0)	1 (4.8)
Penicillins				
Ampicillin	218 (95.6)	147 (82.1)	42 (82.4)	13 (61.9)
Penicillins + β-lactamase inhibitors				
Amoxicillin-clavulanic acid	96 (42.1)	56 (31.3)	32 (62.7)	8 (38.1)
Antipseudomonal penicillins + β-lactamase inhibitors				
Piperacillin-tazobactam	12 (5.3)	6 (3.4)	2 (3.9)	0
Carbapenems				
Imipenem	168 (73.7)	110 (61.5)	30 (58.8)	7 (33.3)
Meropenem	79 (34.6)	33 (18.4)	16 (31.4)	3 (14.3)
Polymyxins				
Colistin	91 (39.9)	177 (98.9)	19 (37.3)	20 (95.2)
Polymyxin B	11 (4.8)	177 (98.9)	4 (7.8)	20 (95.2)
Monobactams				
Aztreonam	20 (8.8)	16 (8.9)	8 (15.7)	2 (9.5)
Aminoglycosides				
Gentamicin	41 (18.0)	56 (31.3)	7 (13.7)	4 (19.0)
Amikacin	37 (16.2)	24 (13.4)	5 (9.8)	0
Tetracyclines				
Doxycycline	170 (74.6)	174 (97.2)	31 (60.8)	18 (85.7)
Folate pathway inhibitors				
Trimethoprim-sulfamethoxazole	210 (92.1)	158 (88.3)	44 (86.3)	16 (76.2)
Glycylcyclines				
Tigecycline	1 (0.4)	2 (1.1)	0	0
Phenicols				
Chloramphenicol	60 (26.3)	75 (41.9)	6 (11.8)	4 (19.0)

For *Salmonella* isolates from retail chicken meat (*n* = 179), the highest percentage of resistance was observed against colistin and polymyxin B (98.9% each), followed by doxycycline (97.2%), pefloxacin (91.1%), trimethoprim-sulfamethoxazole (88.3%), nalidixic acid and ampicillin (82.7% each), and ciprofloxacin (66.5%). Resistance to imipenem (61.5%) and meropenem (18.4%) was also noticeable. Relatively very low resistance was observed to tigecycline (1.1%), piperacillin-tazobactam (3.4%), cefoxitin (7.3%), and aztreonam (8.9%). Among *Salmonella* isolates from LBM sewage (*n* = 21), the result highlighted that 95.2% of isolates were resistant to colistin and polymyxin B, 85.7% resistant to doxycycline, and 81.0% to pefloxacin. Resistance to imipenem (33.3%) and meropenem (14.3%) was also found to be high. Conversely, resistance to ceftazidime, cefoxitin, and levofloxacin remained very low (4.8%), and none of the isolates were resistant to several β-lactams or tigecycline.

### Prevalence of resistance genes

The results of broad-spectrum β-lactamase genes showed that all the isolates of *E. coli* and *Salmonella* isolated from retail chicken meat and LBM sewage samples were positive for the *bla_TEM* gene. The *bla_SHV* gene was detected in only one (0.4%) *E. coli* isolate from retail chicken meat and was absent in all *Salmonella* isolates. Regarding ESBL-encoding genes, *bla_CTX-M-1* was detected in three (1.3%) *E. coli* isolates from retail chicken meat, two (3.9%) *E. coli* isolates from LBM sewage, and five (2.8%) *Salmonella* isolates from retail chicken meat. The *bla_CTX-M-2* gene was detected in a single (2%) *E. coli* isolate from LBM sewage but was absent in all *Salmonella* isolates (Table 3).

**TABLE 3:** Prevalence of resistance genes in *E. coli* and *Salmonella* isolated from retail chicken meat and live bird market (LBM) sewage samples^*a*^

Resistance genes	No. of isolates (%)			
	Retail chicken meat		LBM sewage	
	*E. coli* (*n* = 228)	*Salmonella* (*n* = 179)	*E. coli* (*n* = 51)	*Salmonella* (*n* = 21)
BSBL-encoding genes				
*bla_TEM*	228 (100.0)	179 (100.0)	51 (100.0)	21 (100.0)
*bla_SHV*	1 (0.4)	0	0	0
ESBL-encoding genes				
*bla_CTX-M-1*	3 (1.3)	5 (2.8)	2 (3.9)	0
*bla_CTX-M-2*	0	0	1 (2.0)	0
PMQR-encoding genes				
*qnrA*	22 (9.6)	20 (11.2)	11 (21.6)	0
*qnrB*	5 (2.2)	4 (2.2)	0	0
*qnrS*	162 (71.1)	34 (19.0)	32 (62.7)	8 (38.1)

^*a*^ *n*, number of isolates; BSBL, broad-spectrum β-lactamase; ESBL, extended-spectrum β-lactamase; and PMQR, plasmid-mediated quinolone resistance.

Among PMQR genes, the *qnrA* gene was detected in 9.6% (22/228) and 21.6% (11/51) of *E. coli* isolates from retail chicken meat and LBM sewage samples, respectively, and in 11.2% (20/179) of *Salmonella* isolates from retail chicken meat (Table 3). The *qnrB* gene was detected in 2.2% of *E. coli* and *Salmonella* isolates from retail chicken meat but was absent in isolates from LBM sewage. In contrast, the *qnrS* gene was simultaneously detected in 71.1% (162/228) and 62.7% (32/51) of *E. coli* isolates from retail chicken meat and LBM sewage, respectively, in 19% (34/179) and 38.1% (8/21) of *Salmonella* isolates from the corresponding sample types.

### Coexistence of different resistance genes

Among *E. coli* isolates from retail chicken meat, seven distinct gene coexistence patterns were identified, while five patterns were observed among isolates from LBM sewage. Most isolates harbored two resistance genes (179 from retail chicken meat and 42 from LBM sewage), whereas only seven isolates from retail chicken meat and two isolates from LBM sewage samples carried three genes (Table 4). Coexistence of *bla_TEM* with *qnrS* was identified as the most prevalent combination in both retail chicken meat (*n* = 155) and LBM sewage (*n* = 30). In addition, three isolates from retail chicken meat and one isolate from LBM sewage contained a set of three genes, namely *bla_TEM + bla_CTX-M-1 + qnrS*.

**TABLE 4:** Coexistence of different resistance genes in *E. coli* and *Salmonella* isolated from retail chicken meat and live bird market (LBM) sewage samples

Source	Patterns of resistance genes	No. of isolates (%)
*E. coli*		
Retail chicken meat	*bla_TEM*	42 (18.4)
	*bla_TEM + qnrA*	21 (9.2)
	*bla_TEM + qnrB*	3 (1.3)
	*bla_TEM + qnrS*	155 (68.1)
	*bla_TEM + bla_SHV + qnrS*	1 (0.4)
	*bla_TEM + bla_CTX-M-1 + qnrS*	3 (1.3)
	*bla_TEM + qnrA + qnrS*	1 (0.4)
	*bla_TEM + qnrB + qnrS*	2 (0.9)
	Total	228 (100)
LBM sewage	*bla_TEM*	7 (13.5)
	*bla_TEM + qnrA*	11 (21.2)
	*bla_TEM + qnrS*	30 (57.7)
	*bla_TEM + bla_CTX-M-1*	1 (1.9)
	*bla_TEM + bla_CTX-M-1 + qnrS*	1 (1.9)
	*bla_TEM + bla_CTX-M-2 + qnrS*	1 (1.9)
	Total	51 (100)
*Salmonella*		
Retail chicken meat	*bla_TEM*	129 (72.0)
	*bla_TEM + qnrA*	12 (6.7)
	*bla_TEM + qnrB*	3 (1.7)
	*bla_TEM + qnrS*	24 (13.4)
	*bla_TEM + bla_CTX-M-1 + qnrA*	1 (0.6)
	*bla_TEM + bla_CTX-M-1 + qnrS*	2 (1.1)
	*bla_TEM + qnrA + qnrS*	5 (2.8)
	*bla_TEM + qnrB + qnrS*	1 (0.6)
	*bla_TEM + bla_CTX-M-1 + qnrA + qnrS*	2 (1.1)
	Total	179 (100)
LBM sewage	*bla_TEM*	13 (61.9)
	*bla_TEM + qnrS*	8 (38.1)
	Total	21 (100)

In *Salmonella*, coexistence of different resistance genes was identified in 50 (27.9%) isolates from retail chicken meat and in 8 (38.1%) isolates from LBM sewage samples. Eight different coexistence patterns were observed among retail chicken meat isolates. Of these, 39 isolates carried two different genes, 9 carried three genes, and only 2 isolates harbored four genes (*bla_TEM*, *bla_CTX-M-1*, *qnrA,* and *qnrS*) (Table 4).

### Associations among antimicrobial resistance phenotypes

Associations among 29 AMR phenotypes were analyzed. As shown in Tables 5 to 7, most resistance phenotypes were significantly associated with at least one other AMR phenotype (*P* ≤ 0.05). In *E. coli* from retail chicken meat, the strongest associations were observed between levofloxacin and ciprofloxacin (OR = 680.8), followed by gatifloxacin and levofloxacin (OR = 242.9), nalidixic acid and levofloxacin (OR = 141.1), and several other fluoroquinolone and β-lactam combinations (Table 5).

**TABLE 5:** Association among antimicrobial resistance phenotypes of *E. coli* isolated from retail chicken meat^*a*^

AMs^*b*^	NA	LEV	NOR	CIP	OFX	GAT	CL	CE	CXM	CEC	CAZ	CRO	CTX	CFM	FEP	FOX	AM	AMC	TPZ	IPM	MEM	CT	PB	ATM	CN	AK	DO	SXT	C
NA																													
LEV	141.1																												
NOR	103.9	44.4																											
CIP	28	680.8	75.9																										
OFX	45.7	105.7	112.7	83.9																									
GAT	-	242.9	88.2	138.9	120.7																								
CL	2.3	2.3	1.4	2.3	2.1	1.8																							
CE	1.5	2.7	1.7	2.7	2.7	2.1	1.9																						
CXM	2.1	2.2	1.5	4.1	2.5	1.8	3.2	3.2																					
CEC	4.2	2.4	1.8	2.2	2.3	2.4	8.7	4.4	3.1																				
CAZ	0.9	0.9	0.9	1.4	1.1	0.9	2.01	2.01	32.1	4.5																			
CRO	1.2	1.9	1.9	2.5	1.9	1.5	0.8	3.7	8.5	2.5	5.2																		
CTX	1.1	1.1	0.8	1.5	1.2	0.9	1.8	1.4	26.1	3	21.5	6.8																	
CFM	1.4	1.9	1.5	3.1	1.4	1.2	1.3	4.4	62.6	3.5	23.9	44.6	14																
FEP	2.1	2.2	1.1	2.4	1.7	1.8	3.2	1.8	24	4.1	69.9	6.2	26.1	33.8															
FOX	-	-	4	-	-	-	2.1	2.1	6.9	6.9	-	10.1	2	8.7	-														
AM	18.9	6.8	12.6	5.1	5.04	8.5	1.1	-	-	-	0.8	-	0.6	-	-	-													
AMC	0.9	1.8	1.6	1.3	2.1	1.6	1.01	2.2	1.6	2.5	0.8	1.6	1.5	1.2	1.1	1.4	-												
TPZ	1.3	3.4	4.2	2.6	2.6	2.7	3.1	4.5	4.8	5.2	4.2	3.3	6.4	4.4	2.7	-	-	17.1											
IPM	2.7	3.3	1.8	2.7	3.2	3.3	1.5	0.9	0.8	0.9	1.4	0.8	1.2	0.6	1.2	-	1.2	1.8	1.8										
MEM	7.4	2.3	4.1	2.1	2.7	2.8	0.9	0.5	0.6	1.2	1.4	1.8	0.9	1.7	0.8	10	2.2	0.7	0.9	8.7									
CT	0.8	1.1	0.7	0.9	0.9	0.9	0.7	1.8	0.4	0.6	0.2	0.8	0.5	0.4	0.2	0.3	1.6	2.2	0.3	1.1	0.2								
PB	0.6	1.1	0.6	1.3	2.3	0.9	0.4	5.5	3.1	0.6	1.1	9.2	0.9	5.1	1.3	-	-	2.5	4.6	0.4	0.7	7.4							
ATM	2.4	2.8	1.5	3	2.1	2.2	1.4	1.4	41.1	2.3	62.5	3.5	23.7	15.7	94.7	-	-	0.6	2.2	1.5	2	0.1	1.04						
CN	2.02	2.3	2.9	1.9	2.4	1.8	0.8	2.2	2.7	2.1	1.7	2.8	1.1	3.4	1.4	2.3	-	1.6	5.2	1.1	0.2	2.5	6.2	1.6					
AK	1.4	0.9	1.03	1.4	0.9	1.1	1.7	0.9	0.3	1.3	0.6	1.1	1.3	1.3	1.1	-	-	0.7	4.1	1.1	1.4	0.5	3.2	0.9	0.7				
DO	3.3	3	3	3.3	3.2	3.3	0.6	2.1	1.6	1.5	1.3	1.7	1.1	2	1.1	-	2	1.4	1.8	1.4	4.5	0.3	3.6	2.03	1.5	1.3			
SXT	3.6	2.1	2.1	2.2	1.7	2.6	0.6	2.1	1.4	1.3	0.4	2.1	0.6	1.03	1.4	-	9.7	1.5	0.9	1.4	0.8	1.4	-	0.8	1.8	1.6	2.6		
C	2.3	-	2.5	2.1	3.8	2.3	1.02	1.7	4.6	2.3	3.5	2.4	3.1	3.3	3.5	2.9	3.3	2.2	6.3	2.1	3.4	0.4	2.5	4.1	1.8	1.2	2.3	1.9	

^*a*^ AMs, antimicrobials, and gray shaded cells were statistically significant (*P* ≤ 0.05); and –, undefined odds ratio.

^*b*^ NA, Nalidixic acid; LEV, Levofloxacin; NOR, Norfloxacin; CIP, Ciprofloxacin; OFX, Ofloxacin; GAT, Gatifloxacin; CL, Cephalexin; CE, Cephradine; CXM, Cefuroxime; CEC, Cefaclor; CAZ, Ceftazidime; CRO, Ceftriaxone; CTX, Cefotaxime; CFM, Cefixime; FEP, Cefepime; FOX, Cefoxitin; AM, Ampicillin; AMC, Amoxicillin-clavulanic acid; TPZ, Pipercillin-tazobactam; IPM, Imipenem; MEM, Meropenem; CT, Colistin; PB, Polymyxin B; ATM, Aztreonam; CN, Gentamicin; AK, Amikacin; DO, Doxycycline; SXT, Trimethoprim-sulfamethoxazole; C, Chloramphenicol.

**TABLE 6:** Association among antimicrobial resistance phenotypes of *Salmonella* isolated from retail chicken meat^*a*^

AMs^*b*^	NA	LEV	NOR	CIP	OFX	GAT	PEF	CL	CE	CXM	CEC	CAZ	CRO	CTX	CFM	FEP	FOX	AM	AMC	TPZ	IPM	MEM	PB	ATM	CN	AK	DO	SXT	C
NA																													
LEV	3.6																												
NOR	21.6	5.2																											
CIP	4.6	-	13.8																										
OFX	11	16.3	9.9	7.4																									
GAT	12.7	19.7	4.7	16.4	20.6																								
PEF	137.8	-	-	2.1	-	-																							
CL	0.9	0.8	1.2	3.2	0.6	1.1	0.3																						
CE	0.9	0.8	1	4	0.5	1.3	0.3	1225																					
CXM	0.4	0.7	1.9	7.7	0.4	1.1	0.1	145.8	85.5																				
CEC	0.7	0.3	0.9	3.4	0.4	0.9	0.2	-	-	56.4																			
CAZ	0.4	0.3	0.7	1.5	0.4	0.8	0.2	29.8	29.8	31.8	30.5																		
CRO	0.4	0.9	0.8	12.6	0.6	2.3	0.1	48.9	48.9	50	42.6	35.4																	
CTX	0.7	1.3	1.5	3.6	0.7	1.1	0.4	63.5	63.5	50	87.3	18.7	52.8																
CFM	0.5	1.1	1.2	3.7	0.6	1.7	0.2	109.5	71.9	91.1	53.7	33.7	164.4	59.8															
FEP	5	0.9	2.5	3.6	0.5	1.5	-	48.9	32.7	50	22	14.8	10.1	21.7	41.1														
FOX	0.1	0.8	0.3	0.8	0.5	1.4	0.2	17.2	17.2	8.5	13.3	18.5	17.4	0.05	16.4	3.7													
AM	8.8	1.7	22.6	9.3	3.8	6.2	1.6	-	-	6.7	-	6.7	-	3.9	7.3	-	2.8												
AMC	1.7	0.9	1.4	6.5	0.9	0.9	0.6	7.5	7.5	4.1	6.6	4.1	7.8	8.3	7	3.1	5.7	-											
TPZ	-	2	9.9	-	1.7	1.5	-	4.1	4.1	3	4.8	1.1	3.8	3.1	2.7	8.1	2.7	-	2.3										
IPM	2.6	11.6	1.1	2.3	2.2	5.8	30.3	0.3	0.4	0.3	0.3	0.3	0.4	0.5	0.3	0.5	0.7	0.6	0.9	1.3									
MEM	8.3	3.7	10.9	4.5	8	4.5	-	0.6	0.5	0.3	0.6	0.5	1	0.8	0.7	0.2	0.8	-	0.8	2.3	1.9								
PB	-	-	-	2	-	-	-	-	-	-	-	-	-	-	-	-	0.1	-	-	-	1.6	-							
ATM	-	0.6	2	3.9	0.5	1.4	-	42.6	25.1	20.2	20.3	10.4	3.9	17.7	25.8	334.3	0.8	-	2.4	2.1	0.5	-	-						
CN	1.4	2.1	3.1	5.3	2	1.7	0.4	1.9	1.9	2.4	2.1	2	3.8	1.7	2.4	0.8	1	18.5	1.5	2.3	0.6	5.6	-	0.5					
AK	0.3	0.8	0.4	2.8	0.2	0.8	0.1	10.1	12.8	9.3	12.7	7.4	11.1	6.9	8.1	2.9	3.2	5.8	2.5	7.2	0.3	0.2	0.1	2.4	3.8				
DO	1.2	-	-	8.4	2.4	-	-	-	-	-	-	-	-	-	-	-	-	20.9	-	-	-	-	-	-	-	-			
SXT	9.8	1	12.9	6.3	2.7	3.4	1.9	-	-	3.9	-	3.9	-	7.7	4.3	-	-	106	-	-	0.8	-	-	-	-	-	5.4		
C	14.1	1.6	4.5	4.5	1.7	1.5	12.5	1.6	1.6	1.1	1.4	0.4	0.8	1.3	1	2.2	0.6	14.8	3.9	2.9	1.5	2.2	0.7	2.5	1.1	0.4	3		

^*a*^ AMs, antimicrobials; gray shaded cells were statistically significant (*P* ≤ 0.05); and –, undefined odds ratio.

^*b*^ NA, Nalidixic acid; LEV, Levofloxacin; NOR, Norfloxacin; CIP, Ciprofloxacin; OFX, Ofloxacin; GAT, Gatifloxacin; PEF, Pefloxacin; CL, Cephalexin; CE, Cephradine; CXM, Cefuroxime; CEC, Cefaclor; CAZ, Ceftazidime; CRO, Ceftriaxone; CTX, Cefotaxime; CFM, Cefixime; FEP, Cefepime; FOX, Cefoxitin; AM, Ampicillin; AMC, Amoxicillin-clavulanic acid; TPZ, Pipercillin-tazobactam; IPM, Imipenem; MEM, Meropenem; PB, Polymyxin B; ATM, Aztreonam; CN, Gentamicin; AK, Amikacin; DO, Doxycycline; SXT, Trimethoprim-sulfamethoxazole; C, Chloramphenicol.

**TABLE 7:** Association among antimicrobial resistance phenotypes of *E. coli* isolated from live bird market (LBM) sewage^*a*^

AMs^*b*^	NA	LEV	NOR	CIP	OFX	GAT	PEF	CL	CE	CXM	CEC	CAZ	CRO	CTX	CFM	FEP	AM	AMC	TPZ	IPM	MEM	CT	PB	ATM	CN	AK	DO	SXT	C
NA																													
LEV	-																												
NOR	55	86.7																											
CIP	26.1	-	87.5																										
OFX	30	-	-	101.2																									
GAT	65.7	-	286	598	-																								
PEF	-	-	-	-	-	-																							
CL	5	10.5	9.5	8.7	6	8.7	-																						
CE	1.5	1.8	1.7	1.5	1	1.5	0.4	10.4																					
CXM	0.9	1.9	1.8	3.3	1.1	1.6	-	3.1	4.8																				
CEC	2.4	5.1	11.8	10.7	7.3	10.7	0.3	13.3	1.7	5.9																			
CAZ	2.2	4.4	4.1	3.7	2.6	3.7	-	8.4	20.5	32.3	25.2																		
CRO	1.2	1.4	1.3	1.2	0.8	1.2	0.3	5.9	7.6	13.3	4.6	-																	
CTX	1.8	1.4	1.7	1.4	0.8	1.4	1.4	4.4	2	-	2.8	-	-																
CFM	0.7	1.4	1.3	1.1	0.8	1.1	-	4	16	28	3.3	44	20.5	9.1															
FEP	0.5	1	0.9	1.6	0.6	0.8	-	3.1	4.8	258	2.5	32.3	13.3	-	28														
AM	3.6	8	8.8	9.7	5.7	9.7	11.7	-	0.2	1.3	1.6	0.2	0.3	0.9	1.1	0.5													
AMC	1.5	3.2	3.6	4.1	4.8	4.1	3.6	1.6	0.4	1.6	2	0.2	0.3	1.9	1.2	1.6	22.5												
TPZ	0.7	1.3	1.2	1.1	0.8	1.1	0.04	7.2	-	-	-	-	-	1.5	-	-	-	-											
IPM	6.6	10.4	7.3	5.5	6.9	8.5	-	1.9	7.3	5	1.2	-	2.4	1.8	-	1.9	2	2.1	0.7										
MEM	20	12.5	50.6	9.5	25.4	17.5	-	3.6	1.1	1.8	2.6	2.4	1.4	5.5	1.1	0.9	4.4	6.6	2.3	-									
CT	1.9	1.9	1.2	1.4	1.2	1.4	1.2	2.6	4.5	1.3	1.9	5.8	3.5	0.8	4	1.3	0.7	0.5	1.7	2.8	0.3								
PB	2.2	4.4	4.1	3.7	2.6	3.7	-	2.3	1.6	2.3	1.9	4.9	1.9	4.8	2.8	2.3	0.6	1.9	15.3	-	2.4	-							
ATM	0.7	0.8	0.7	1.2	0.8	0.6	-	2.5	3.7	126	2.1	-	22.2	14.5	20.5	126	1.6	1	-	2.4	0.7	3.5	1.9						
CN	5	4	0.9	3.3	2.3	3.3	-	1.1	-	-	0.9	-	0.9	0.5	1.3	-	-	1.6	-	0.9	0.9	2.6	-	0.9					
AK	1.1	0.9	0.8	0.7	3.7	0.7	-	1.7	-	-	1.4	-	-	0.9	-	-	0.8	0.9	11.3	1.1	1.5	1.1	3.6	1.4	-				
DO	12.5	16.4	46.4	11.9	13.7	22	-	4.6	0.8	0.8	5.5	2	0.6	2.2	1.3	0.4	20	7.7	0.6	3.7	-	0.8	2	0.3	0.8	0.4			
SXT	4.4	-	-	-	9.5	-	17.2	-	0.1	0.9	-	0.4	0.4	0.9	0.8	0.3	-	14.3	-	2.1	-	0.4	0.4	1.1	-	0.6	-		
C	4	8.2	7.5	-	4.8	6.8	-	1.3	0.9	4	3.3	-	-	0.7	1.6	1.3	-	3.3	-	0.7	1.1	1.8	-	1.1	10.3	-	1.3	-	

^*a*^ AMs, antimicrobials; gray shaded cells were statistically significant (*P* ≤ 0.05); and –, undefined odds ratio.

^*b*^ NA, Nalidixic acid; LEV, Levofloxacin; NOR, Norfloxacin; CIP, Ciprofloxacin; OFX, Ofloxacin; GAT, Gatifloxacin; PEF, Pefloxacin; CL, Cephalexin; CE, Cephradine; CXM, Cefuroxime; CEC, Cefaclor; CAZ, Ceftazidime; CRO, Ceftriaxone; CTX, Cefotaxime; CFM, Cefixime; FEP, Cefepime; FOX, Cefoxitin; AM, Ampicillin; AMC, Amoxicillin-clavulanic acid; TPZ, Pipercillin-tazobactam; IPM, Imipenem; MEM, Meropenem; PB, Polymyxin B; ATM, Aztreonam; CN, Gentamicin; AK, Amikacin; DO, Doxycycline; SXT, Trimethoprim-sulfamethoxazole; C, Chloramphenicol.

In *Salmonella* from retail chicken meat, strong associations were observed between cephalexin and cephradine (OR = 1,225), cefepime and aztreonam (OR = 334.3), ceftriaxone and cefixime (OR = 164.4), cefuroxime and cephalexin (OR = 145.8), nalidixic acid and pefloxacin (OR = 137.8), cefixime and cephalexin (OR = 109.5), and ampicillin and trimethoprim-sulfamethoxazole (OR = 106.1) (Table 6). Among isolates from LBM sewage, strong associations in *E. coli* were observed between ciprofloxacin and gatifloxacin (OR = 598), norfloxacin and gatifloxacin (OR = 286), cefuroxime and cefepime (OR = 258), aztreonam and cefuroxime (OR = 126), ampicillin and aztreonam (OR = 126), and ciprofloxacin and ofloxacin (OR = 101.2) (Table 7). No significant associations between different antimicrobial resistance phenotypes were detected among *Salmonella* isolates from LBM sewage.

### Associations among antimicrobial resistance genes

Among *E. coli* isolates from retail chicken meat, only PMQR-encoding genes, mainly *qnrA* and *qnrS,* were significantly associated with each other, showing a negative association *(*OR *=* 0.013, *P <* 0.001) (Table 8). In *Salmonella*, positive associations were identified between *bla_CTX-M-1* and *qnrA* (OR = 13.9*, P =* 0.006) and between *bla_CTX-M-1* and *qnrS* (OR *=* 19.2*, P =* 0.009)*.* No significant associations were observed among other ARGs, such as *bla_TEM, bla_SHV*, and *qnrB* genes in the case of retail chicken meat and *bla_TEM, bla_CTX-M-1, bla_CTX-M-2, qnrA,* and *qnrS* genes in the case of LBM sewage samples.

**TABLE 8:** Pairwise statistical correlation between antimicrobial resistance genes (β-lactamase- and PMQR-encoding genes) in *E. coli* (*n* = 228) and *Salmonella* (*n* = 179) isolated from retail chicken meat^*b*^

Types of bacteria	ARGs	Associated ARGs	OR	95% CI	*P*-value^*a*^
*E. coli*	*qnrA*	*qnrS*	0.014	0.002–0.1	<0.001
	*qnrS*	*qnrA*	0.014	0.002–0.1	<0.001
*Salmonella*	*bla_CTX-M-1*	*qnrA*	13.9	2.2–88.8	0.006
		*qnrS*	19.2	2.1–177.9	0.009
	*qnrA*	*bla_CTX-M-1*	13.9	2.2–88.8	0.006
	*qnrS*	*bla_CTX-M-1*	19.2	2.1–177.9	0.009

^*a*^ Only statistically significant (*P* ≤ 0.05) associations are shown.

^*b*^ ARGs, antimicrobial resistance genes; PMQR, plasmid-mediated quinolone resistance; OR, odds ratio; and CI, confidence interval.

### Associations between phenotypes and genotypes of antimicrobial resistance

In *E. coli* isolates from retail chicken meat, resistance to nalidixic acid, norfloxacin, and gatifloxacin was not exclusively associated with the presence of PMQR genes, as both resistant and susceptible isolates carried the *qnrS* gene. These phenotypes (both resistant and susceptible) were negatively associated with the presence of the *qnrS* gene (OR <1). Conversely, resistance to several non-fluoroquinolone antimicrobials, including cephalosporins, gentamicin, trimethoprim–sulfamethoxazole, and aztreonam, was associated with the presence of PMQR genes (either *qnrA* or *qnrB* or *qnrS*). Among these antimicrobials, cefoxitin resistance was strongly associated with the presence of the *qnrB* gene (OR = 36.5, *P* = 0.001), followed by cefixime (OR = 13.0, *P* = 0.006) and cefaclor (OR = 10.4, *P* = 0.01) resistance (Table 9). On the other hand, a negative association (OR <1) was observed among trimethoprim-sulfamethoxazole with *qnrA* gene. Resistance to cephalosporins was also strongly associated with the ESBL-encoding gene *bla_CTX-M-1*, particularly for cefepime (OR = 28, *P* = 0.008) and ceftazidime (OR = 24.5, *P* = 0.01). On the other hand, resistance to aztreonam (an antimicrobial other than cephalosporins) also contained the ESBL-encoding gene *bla_CTX-M-1.*

**TABLE 9:** Association between antimicrobial resistance phenotypes and antimicrobial resistance genes (β-lactamase- and PMQR-encoding genes) of *E. coli* isolated from retail chicken meat (*n* = 228) and LBM sewage samples (*n* = 51)^*b*^^,*c*^

Antimicrobials	*N*_P_	ARGs	P+/G+	P+/G−	P−/G+	P−/G−	*N*_G_	OR	95% CI	*P*-value^*a*^
Retail chicken meat										
Nalidixic acid	182	*qnrS*	118	64	44	2	162	0.1	0.02–0.4	0.001
Norfloxacin	128	*qnrS*	80	48	82	18	162	0.4	0.2–0.7	0.002
Gatifloxacin	150	*qnrS*	100	50	62	16	162	0.5	0.3–1.1	0.05
Cephalexin	45	*qnrA*	8	37	14	169	22	2.6	1.0–6.7	0.05
Cefuroxime	17	*qnrB*	2	15	3	208	5	9.2	1.4–59.6	0.02
Cefaclor	31	*bla_CTX-M-1*	2	29	1	196	3	13.5	1.2–153.8	0.04
	31	*qnrB*	3	28	2	195	5	10.4	1.7–65.3	0.01
Ceftazidime	19	*bla_CTX-M-1*	2	17	1	208	3	24.5	2.1–283.8	0.01
	19	*qnrA*	5	14	17	192	22	4.0	1.3–12.6	0.02
Ceftriaxone	23	*qnrB*	2	21	3	202	5	6.4	1.0–40.6	0.05
Cefotaxime	46	*qnrB*	3	43	2	180	5	6.3	1.0–38.7	0.05
Cefixime	26	*bla_CTX-M-1*	2	24	1	201	3	16.8	1.5–191.7	0.02
	26	*qnrB*	3	23	2	200	5	13.0	2.1–82.2	0.006
Cefepime	17	*bla_CTX-M-1*	2	15	1	210	3	28.0	2.4–326.7	0.008
Cefoxitin	6	*qnrB*	2	4	3	219	5	36.5	4.7–281.9	0.001
Gentamicin	41	*qnrS*	18	23	144	43	162	0.2	0.1–0.5	<0.001
Trimethoprim-sulfamethoxazole	210	*qnrA*	15	195	7	11	22	0.1	0.04–0.4	<0.001
Aztreonam	20	*bla_CTX-M-1*	2	18	1	207	3	23.0	2.1–266.1	0.01
LBM sewage										
Trimethoprim-sulfamethoxazole	44	*qnrA*	6	38	5	2	11	0.06	0.01–0.4	0.003
		*qnrS*	31	13	1	6	32	14.3	1.6–130.9	0.02
Ampicillin	42	*qnrA*	6	36	5	4	11	0.1	0.03–0.6	0.01
		*qnrS*	30	12	2	7	32	8.8	1.6–48.3	0.01

^*a*^ Only statistically significant (*P* ≤ 0.05) associations are shown.

^*b*^ ARGs, antimicrobial resistance genes; PMQR, plasmid-mediated quinolone resistance; OR, odds ratio; and CI, confidence interval.

^*c*^ *N*_P_, number of isolates expressing phenotypic resistance to the indicated antimicrobials. P+/G+, number of phenotypically resistant isolates (P+) with resistance genes (G+) for antimicrobials identified. P+/G−, number of phenotypically resistant isolates (P+) with no resistance genes (G−) for antimicrobials identified. P−/G+, number of phenotypically susceptible isolates (P−) with resistance genes (G+) for antimicrobials identified. P−/G−, number of phenotypically susceptible isolates (P−) with no resistance genes (G−) for antimicrobials identified. *N*_G_, number of isolates carrying the indicated resistance gene.

In LBM sewage isolates, trimethoprim-sulfamethoxazole and ampicillin resistance in *E. coli* was strongly associated with the presence of the *qnrS* gene. No significant associations were observed for *bla_TEM* and *bla_SHV* with any of the phenotypic antimicrobial resistance.

*In Salmonella* isolates from retail chicken meat, several strong associations were identified between resistance phenotypes and corresponding genes, including aztreonam resistance with *bla_CTX-M-1* gene (OR = 18.6, *P =* 0.002) and ceftriaxone resistance with *qnrA* gene (OR = 16.4*, P <* 0.001), cefepime resistance with *bla_CTX-M-1* gene (OR = 12.2*, P =* 0.008), and cefuroxime resistance with *qnrA g*ene (OR = 10.9, *P <* 0.001) (Table 10). Nalidixic acid and pefloxacin were negatively associated (OR <1) with the *qnrA* gene and colistin and polymyxin B with the *qnrB* gene. Among *Salmonella* isolates from LBM sewage, a strong association was detected between amoxicillin-clavulanic acid resistance and *qnrS* gene (OR = 84, *P =* 0.003). No associations with any of the phenotypic antimicrobial resistance were observed for *bla_TEM*.

**TABLE 10:** Association between antimicrobial resistance phenotypes and antimicrobial resistance genes (β-lactamase- and PMQR-encoding genes) of *Salmonella* isolated from retail chicken meat (*n* = 179) and live bird market (LBM) sewage (*n* = 21) samples^*b*^^,*c*^

Antimicrobials	*N*_P_	ARGs	P+/G+	P+/G−	P−/G+	P−/G−	*N*_G_	OR	95% CI	*P*-value^*a*^
Retail chicken meat										
Nalidixic acid	148	*qnrA*	13	135	7	24	20	0.3	0.1–0.9	0.03
Ciprofloxacin	119	*qnrA*	18	101	2	58	20	5.2	1.2–23.1	0.03
Gatifloxacin	45	*qnrA*	9	36	11	123	20	2.8	1.1–7.3	0.04
Pefloxacin	163	*qnrA*	13	150	7	9	20	0.1	0.04–0.3	<0.001
Norfloxacin	63	*qnrS*	18	45	16	100	34	2.5	1.2–5.3	0.02
Cephalexin	37	*qnrA*	12	25	8	134	20	8.0	2.9–21.7	<0.001
Cephradine	37	*qnrA*	12	25	8	134	20	8.0	2.9–21.7	<0.001
Cefuroxime	27	*bla_CTX-M-1*	3	24	2	150	5	9.4	1.5–59.1	0.02
		*qnrA*	11	16	9	143	20	10.9	3.9–30.3	<0.001
		*qnrS*	10	17	24	128	34	3.1	1.3–7.7	0.01
Cefaclor	33	*bla_CTX-M-1*	3	30	2	144	5	7.2	1.2–44.9	0.04
		*qnrA*	11	22	9	137	20	7.6	2.8–20.5	<0.001
Ceftazidime	27	*qnrA*	10	17	10	142	20	8.4	3.0–22.9	<0.001
Cefotaxime	45	*qnrA*	11	34	9	125	20	4.5	1.7–11.7	0.002
Ceftriaxone	22	*qnrA*	11	11	9	148	20	16.4	5.6–48.1	<0.001
Cefixime	29	*bla_CTX-M-1*	3	26	2	148	5	8.5	1.4–53.6	0.02
		*qnrA*	11	18	9	141	20	9.6	3.5–26.2	<0.001
Cefepime	22	*bla_CTX-M-1*	3	19	2	155	5	12.2	1.9–77.9	0.008
		*qnrS*	8	14	26	131	34	2.9	1.1–7.6	0.03
Aztreonam	16	*bla_CTX-M-1*	3	13	2	161	5	18.6	2.8–121.3	0.002
Cefoxitin	13	*qnrA*	4	9	16	150	20	4.2	1.2–15.1	0.03
Piperacillin-tazobactam	6	*qnrS*	4	2	30	143	34	9.5	1.7–54.4	0.01
Gentamicin	56	*qnrA*	12	44	8	115	20	3.9	1.5–10.2	0.005
Amikacin	24	*qnrA*	9	15	11	144	20	7.9	2.8–21.9	<0.001
Colistin	177	*qnrB*	3	174	1	1	4	0.02	0.001–0.3	0.008
Polymyxin B	177	*qnrB*	3	174	1	1	4	0.02	0.001–0.3	0.008
Chloramphenicol	75	*qnrS*	21	54	13	91	34	2.7	1.3–5.9	0.01
LBM sewage										
Amoxicillin-clavulanic acid	8	*qnrS*	7	1	1	12	8	84	4.5–1,564.3	0.003

^*a*^ Only statistically significant (*P* ≤ 0.05) associations are shown.

^*b*^ ARGs, antimicrobial resistance genes; PMQR, plasmid-mediated quinolone resistance; OR, odds ratio; CI, confidence interval.

^*c*^ *N*_P_, number of isolates expressing phenotypic resistance to the indicated antimicrobials. P+/G+, number of phenotypically resistant isolates (P+) with resistance genes (G+) for antimicrobials identified. P+/G−, number of phenotypically resistant isolates (P+) with no resistance genes (G−) for antimicrobials identified. P−/G+, number of phenotypically susceptible isolates (P−) with resistance genes (G+) for antimicrobials identified. P−/G−, number of phenotypically susceptible isolates (P−) with no resistance genes (G−) for antimicrobials identified. *N*_G_, number of isolates carrying the indicated resistance gene.

### Nucleotide and amino acid analysis of the *E. coli* isolates

Some nucleotide substitutions were observed in two of the sequences (one isolate from LBM sewage and one isolate from retail chicken meat). Among four nucleotide substitutions, one isolate from the LBM sewage sample had substitutions at position 87 (T > C) and 285 (C > T). Two substitutions were common in these two isolates, i.e., at positions 309 (T > A) and 363 (A > G). However, there was no amino acid substitution (Fig. S1 and S2).

### Nucleotide and amino acid analysis of the PMQR genes

**Fig 3:**
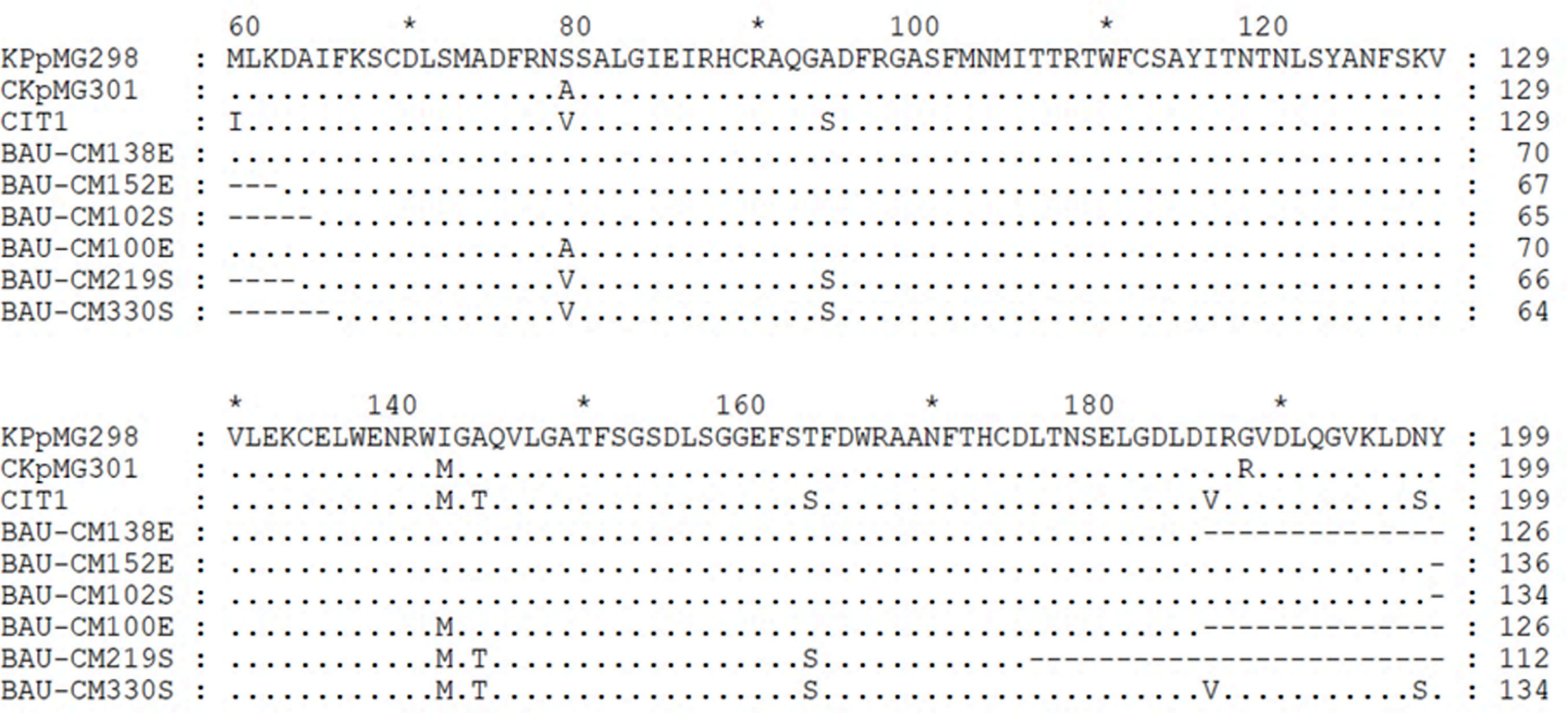
Comparative alignment for amino acid comparison of six *qnrB* genes. The amino acid sequences of KPpMG298 (GenBank accession no.: NG_050469.1), CKpMG301 (GenBank accession no.: DQ351242.1), and CIT1 (GenBank accession no.: NG_050472.2) at the top were used as reference sequences. Identity to the top sequence is marked by a dot (.), and difference is presented by the abbreviated letter of amino acids.

No nucleotide and amino acid substitutions were observed among *qnrA* and *qnrS* sequences. In contrast, variations were detected within *qnrB* sequences. Comparative analysis against reference strains KPpMG298 (*qnrB1*), CKpMG301 (*qnrB2*), and CIT1 (*qnrB12*) (GenBank accession numbers: NG_050469.1, DQ351242.1, and NG_050472.2, respectively; positions: 60–199) revealed amino acid substitutions at positions 79 (S > A and V) and 142 (I > M) in one *E. coli* and two *Salmonella* isolates. These two *Salmonella* isolates had another amino acid substitution at positions 94 (A > S), 144 (A > T), and 163 (T > S). Amino acid substitutions at positions 186 (I > V) and 198 (*N* > S) were also observed in one *Salmonella* isolate. It is noted that these two isolates had no amino acid substitution with the *qnrB12* reference sequence (Fig. 3).

### Phylogenetic analysis of *E. coli*

**Fig 4:**
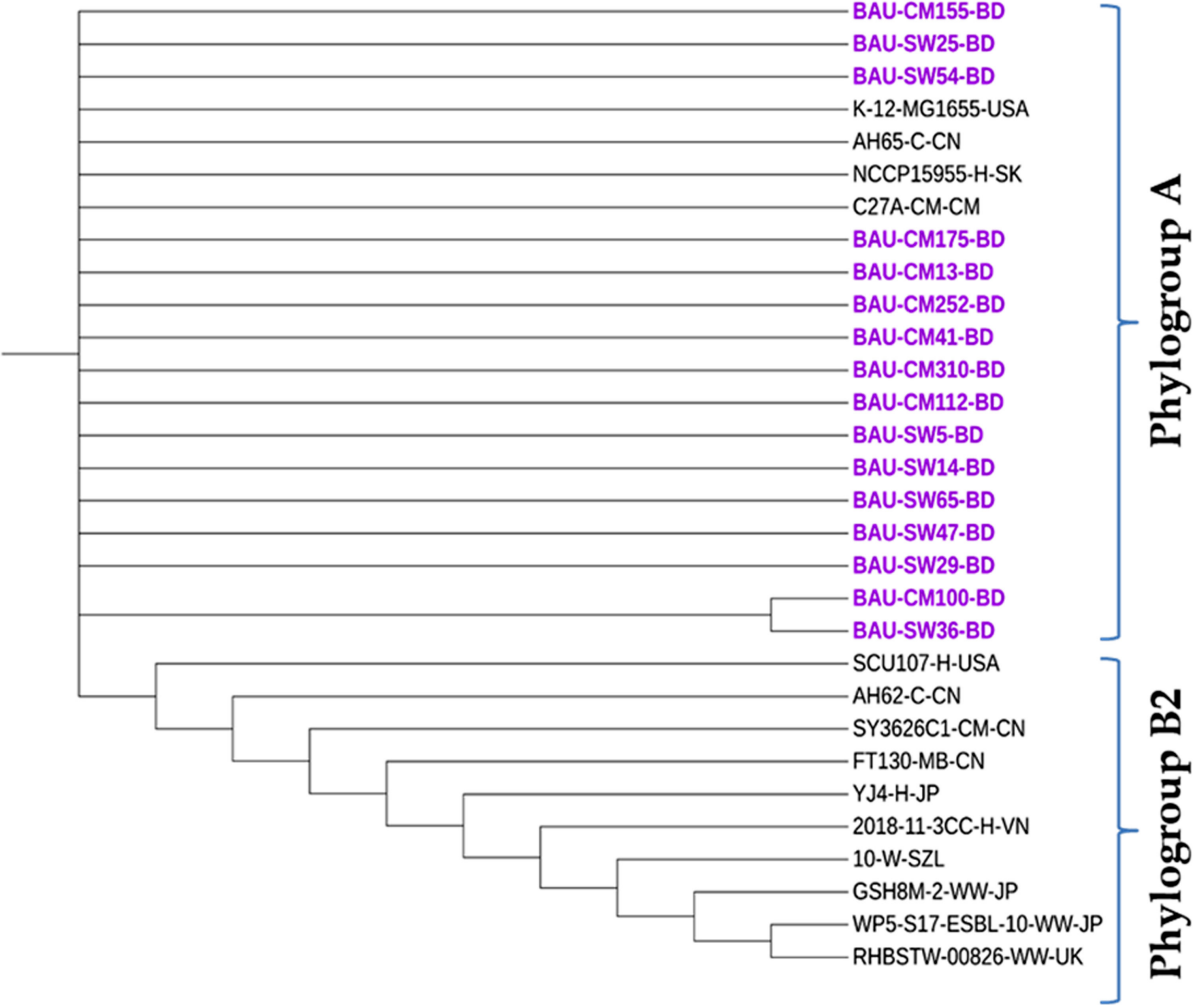
Phylogenetic tree of the *malB* promoter gene of *E. coli* isolates (*n* = 16) from retail chicken meat and LBM sewage and *E. coli* strains retrieved from GenBank, generated using the neighbor-joining method in MEGA X. Bootstrap support values (1,000 replicates) are not shown next to branches. The consensus tree was drawn by a cutoff value of 50%.

Based on phylogenetic analysis of *malB* promoter gene sequences, the *E. coli* isolates were grouped into two phylogroups: phylogroup A and phylogroup B2 (Fig. 4). It was observed that all studied *E. coli* isolates belonged to phylogroup A and were found to be closely related to the sequence of reference strain K-12-MG1655 (accession no.: NC_000913.3) and also the strains reported earlier from China, South Korea, and Cambodia.

### Phylogenetic analysis of *Salmonella*

**Fig 5:**
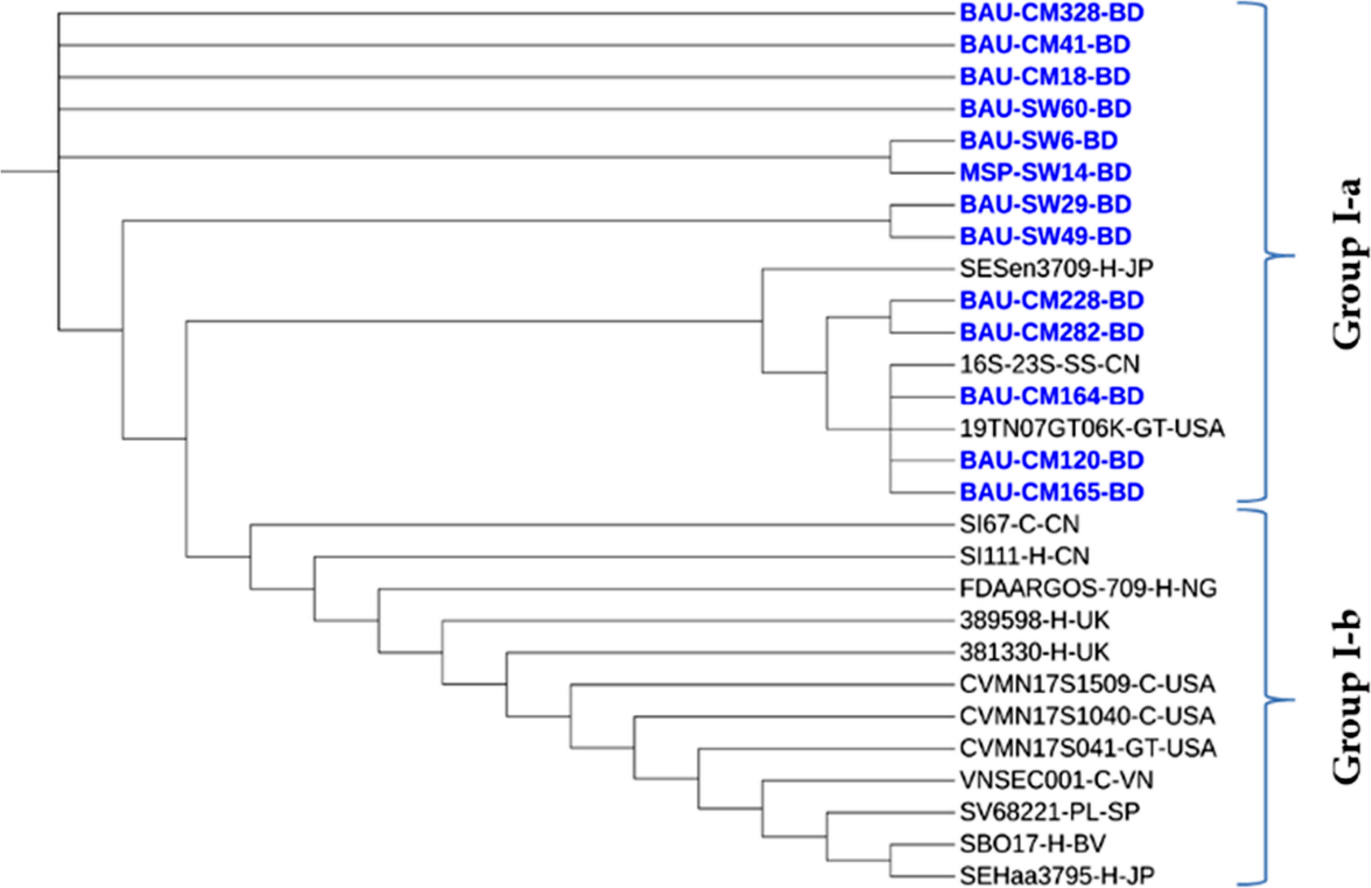
Phylogenetic tree of the *ITS* gene of *Salmonella* isolates (*n* = 13) from retail chicken meat and LBM sewage and *Salmonella* spp. strains retrieved from GenBank generated using the neighbor-joining method in MEGA X. Bootstrap support values (1,000 replicates) are not shown next to branches. The consensus tree was drawn by a cutoff value of 50%.

Phylogenetic reconstruction based on *ITS* gene sequences showed that all study isolates clustered within *Salmonella enterica* subspecies *enterica* (group I-a), closely related to strains from Japan and the United States (Fig. 5).

### Phylogenetic analysis of β-lactamase genes

**Fig 6:**
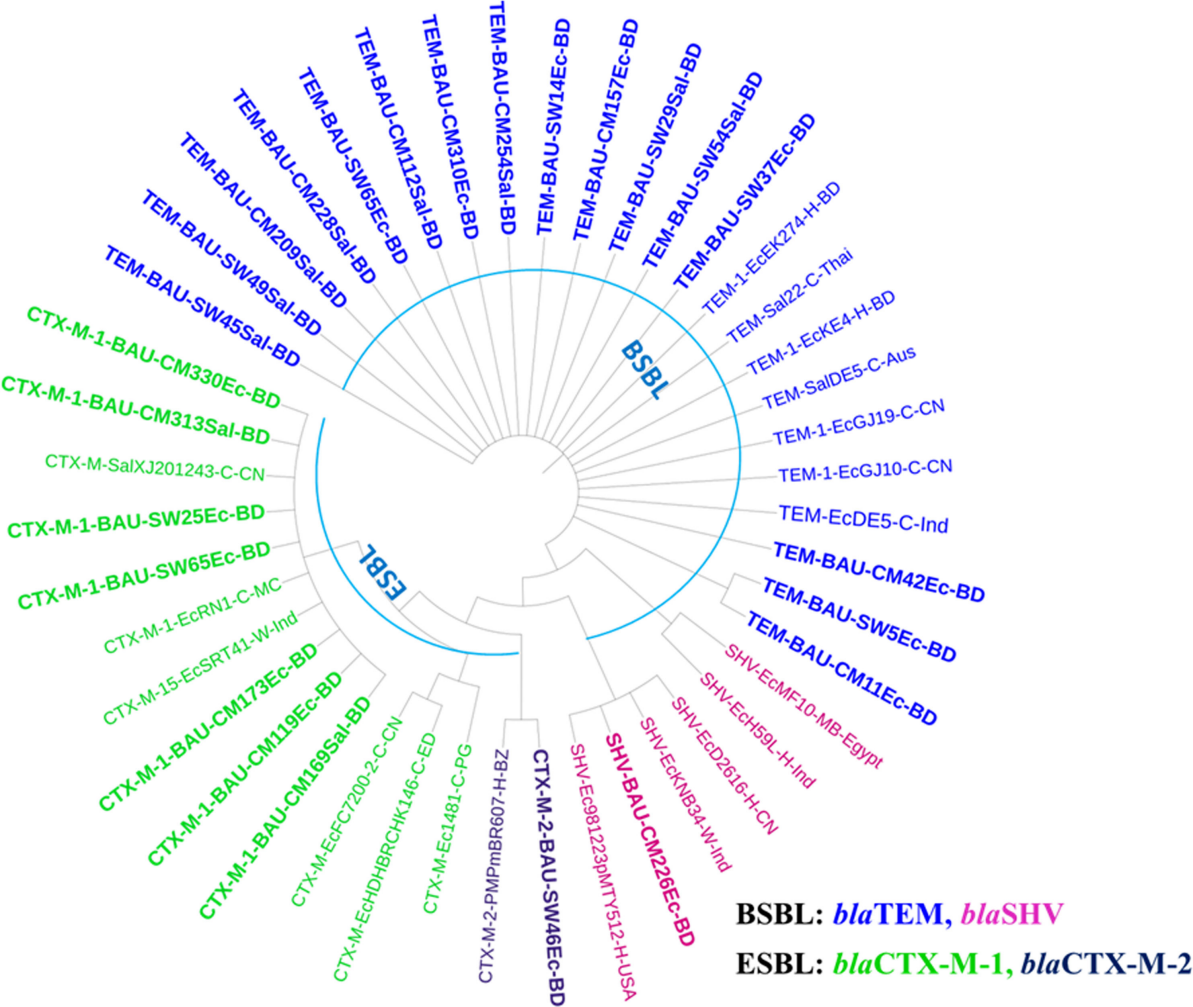
Phylogenetic tree of β-lactamase genes (*n* = 25) generated using the neighbor-joining method in MEGA X. The consensus tree was drawn by a cutoff value of 50%.

Phylogenetic analysis of *bla_TEM*, *bla_SHV*, *bla_CTX-M-1*, and *bla_CTX-M-2* sequences demonstrated clear clustering into TEM, SHV, and CTX-M families of class A β-lactamases. TEM and SHV formed the broad-spectrum β-lactamase group, whereas *CTX-M-1* and *CTX-M-2* formed the ESBL group (Fig. 6). However, we did not observe any nucleotide and amino acid substitution among the sequences of the same family.

### Phylogenetic analysis of PMQR genes

**Fig 7:**
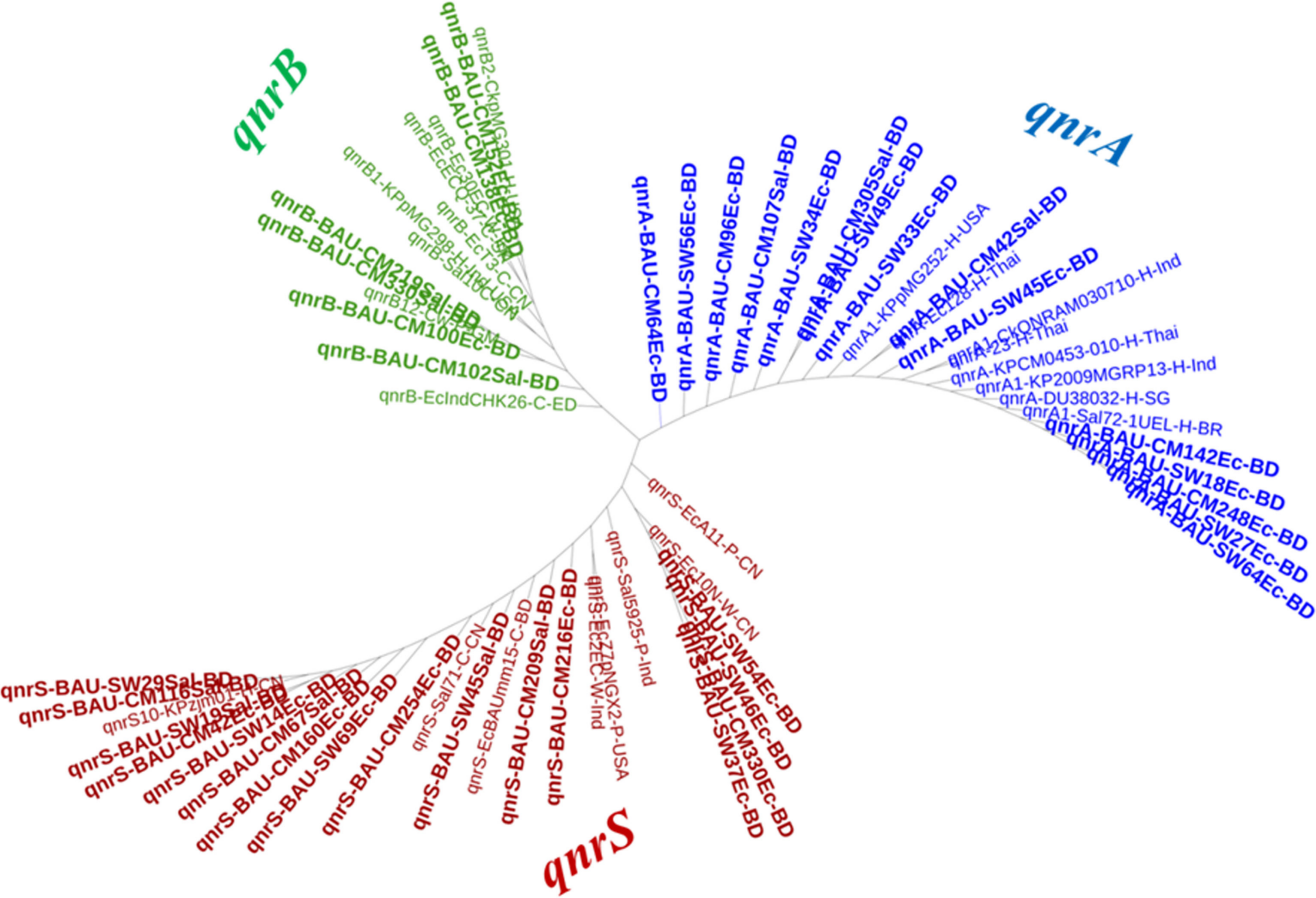
Phylogenetic tree of PMQR genes (*n* = 37) generated using the neighbor-joining method in MEGA X. The consensus tree was drawn by a cutoff value of 50%.

The resulting tree showed clear clustering into three distinct groups corresponding to *qnrA*, *qnrB*, and *qnrS*, highlighting both genetic homogeneity within groups and divergence between them (Fig. 7).

## DISCUSSION

The current study documented comprehensive findings on the wide distribution of AMR *E. coli* and *Salmonella* carrying BSBL-, ESBL-, and PMQR-encoding genes in retail chicken meat and LBM sewage samples collected from 64 LBMs in 32 upazilas across 16 districts representing all 8 divisions of Bangladesh. Significant correlations were observed between phenotypic resistance and the presence of resistance genes. Overall, in retail chicken meat, the prevalence of *E. coli* was higher (71%) than *Salmonella* (56%). The prevalence of *E. coli* in this study exceeds earlier reports from Bangladesh (49%–53%) and is also higher than findings from Brazil (58.6%), Nepal (54.5%), Korea (50.5%), and Pakistan (42%), highlighting a substantial public health concern (3, 15, 16, 33–35). Furthermore, the prevalence of *E. coli* was also higher (80%) in LBM sewage samples, aligning with a previous report of 85% in untreated sewage from Mymensingh district (18). In contrast, the prevalence of *Salmonella* was comparatively lower in both retail chicken meat (55.9%) and LBM sewage (32.8%) samples than in earlier studies in Bangladesh, which reported 63%–70% in retail chicken meat and about 95% in LBM sewage (16, 18, 36–38). Studies in other countries have documented the prevalence of *Salmonella* in retail chicken meat, ranging from 60% in Nepal to 100% in Malaysia (39, 40). Differences in prevalence can be explained by the differences in the locality, sampling season, sample numbers, method of sampling, and the variations in the isolation methods employed to detect the bacteria in individual studies (3). Importantly, contamination of chicken meat with *E. coli* and *Salmonella* often arises from poor hygiene, post-processing contamination through repeated washing with dirty water, improperly prepared/cooked meat, or inadequate hygiene practices among slaughterers and LBM workers.

In this study, phylogenetic analysis of the *malB* promoter gene sequences showed that all *E. coli* isolates belonged to phylogenetic group A, which is associated with diseases in chicken (41). Similar findings have been reported in Japan and the United States, where avian pathogenic *E. coli* isolates were predominantly from phylogenetic group A (42, 43).

Phylogenetic analysis of *Salmonella ITS* gene sequences from retail chicken meat and LBM sewage samples placed them within a single subclade of *Salmonella enterica* subspecies *enterica*, indicating close relatedness. Comparable studies have also shown clustering of different *Salmonella* species (44).

This study revealed alarmingly high levels of AMR in both *E. coli* and *Salmonella* isolated from retail chicken meat and LBM sewage. Contamination of retail chicken meat likely arises through multiple points along the farm-to-market continuum, including antimicrobial use at the farm level, fecal contamination during transport and holding, and extensive cross-contamination during slaughter, defeathering, evisceration, and retail handling in LBMs. All isolates exhibited resistance to at least one antimicrobial agent, with MDR detected in nearly all *E. coli* (99.1% from retail chicken meat and 92.2% from LBM sewage) and *Salmonella* isolates (99.4% and 100%, respectively). These findings highlight the widespread and entrenched nature of resistance in poultry-associated pathogens in Bangladesh (45, 46).

Resistance to doxycycline, ampicillin, and trimethoprim-sulfamethoxazole was extremely high in both *E. coli* (74.6%–95.6%) and *Salmonella* (82.7%–97.2%) from retail chicken meat and LBM sewage, aligning with previous reports from Bangladesh (16, 47, 48). High fluoroquinolone resistance was also observed in *E. coli* and *Salmonella*, with 67% of isolates from retail chicken meat resistant to ciprofloxacin, a sharp increase compared to earlier studies (7%–44% in *E. coli*; 9%–20% in *Salmonella*) (15, 16, 33, 37), likely due to unregulated use of ciprofloxacin in poultry production in Bangladesh. Notably, both pathogens retained low resistance to tigecycline, cefoxitin, and piperacillin-tazobactam, although their clinical utility in poultry-associated infections is limited.

In this study, *E. coli* exhibited unexpectedly high levels of carbapenem resistance in isolates from both retail chicken meat and LBM sewage. Although carbapenem resistance was also detected in *Salmonella*, it occurred at consistently lower levels compared to *E. coli*. Nevertheless, the resistance levels observed for both pathogens exceeded those previously reported, including 45.8%–46.7% in Korea (35, 49), 6% in Pakistan (50), and 3.5% in India (51) for *E. coli*, as well as 1.8% in South Korea (52) and 5.2% in China (53) for *Salmonella*. These elevated resistance levels in both pathogens may be due to co-selection and/or cross-resistance driven by the use of other antimicrobials, particularly doxycycline and trimethoprim-sulfamethoxazole (54).

In this study, a remarkable and concerning finding was the exceptionally high resistance of *Salmonella* isolated from retail chicken meat to last-resort antimicrobials, specifically colistin and polymyxin B, with prevalence approaching 98.9%. This resistance pattern contrasts sharply with that observed in *E. coli*, where such extensive resistance to these critical antimicrobials was not detected. Colistin and polymyxin B are classified as “Reserve” antibiotics under the WHO AWaRe framework and are often considered the final therapeutic option for MDR gram-negative infections, particularly carbapenem-resistant Enterobacteriaceae (55). The emergence of colistin resistance in *Salmonella*, therefore, poses a significant threat to both veterinary and human medicine.

Similar resistance profiles detected in LBM sewage indicate continuous circulation of resistant bacteria within poultry-associated environments, likely facilitated by fecal contamination, shared water systems, slaughter surfaces, and inadequate sanitation, which enable persistence and cross-contamination along the production-to-market chain. These findings suggest that poultry environments sustain and disseminate distinct resistance traits, with *E. coli* acting as an important reservoir for carbapenem resistance and *Salmonella* for colistin resistance, underscoring the urgent need for improved hygiene, targeted surveillance, and stricter control of antimicrobial use in poultry production.

β-lactamase activity in gram-negative bacteria is the major cause of resistance to β-lactam antibiotics. In general, β-lactamases in the Enterobacteriaceae are primarily TEM and CTX-M enzymes, which confer resistance to β-lactam penicillins and cephalosporins (56). The *bla*_TEM gene was the most prevalent BSBL-encoding gene found in all the isolates of *E. coli* and *Salmonella* from retail chicken meat and LBM sewage samples, which is significantly higher in comparison to 6.3% and 25.4% (*bla_TEM* gene) in *E. coli* and *Salmonella* spp. isolated from retail chicken meat in Germany and India, respectively (57, 58). In Bangladesh, the *bla_TEM* gene was previously detected in 41% of *E. coli* and 35% of *Salmonella* from water samples (12, 59). Another BSBL-encoding gene, the *bla_SHV*, was isolated from only one *E. coli* isolate from retail chicken meat samples, which is in line with a previous investigation conducted in Turkey (60). The ESBL-encoding gene *bla_CTX-M-1* was detected in 1.3% of *E. coli* and 2.8% of *Salmonella* isolates from retail chicken meat. In contrast, 41.9% of *E. coli* and 100% of *Salmonella* isolates originating from retail chicken meat, harbored *bla_CTXM-1* gene in Turkey and South Korea, respectively (60, 61). The *bla_CTX-M-1* and *bla_CTX-M-2* ESBL-encoding genes were also detected in 3.9% and 2%, respectively, of *E. coli* isolates from LBM sewage samples, in contrast with previous reports describing these genes as more frequent and present sporadically in environmental samples in Pakistan (50). However, these two genes were not observed in any *Salmonella* spp. isolates from LBM sewage samples.

It has been previously reported that PMQR determinants encoded by *qnrA*, *qnrB*, and *qnrS* genes confer low-level resistance to quinolones and/or fluoroquinolones (62). PMQR genes (*qnrA*, *qnrB*, and *qnrS*) have been found in a variety of Enterobacteriaceae, especially *E. coli* and *Salmonella*. The current study showed increased occurrence of *qnr* genes, particularly the *qnrS* gene, in *E. coli* and *Salmonella* from retail chicken meat and LBM sewage samples. This result contrasts with those of earlier studies conducted in Korea, India, and Bangladesh, where low incidences of the *qnrS* gene in *E. coli* and *Salmonella* recovered from retail chicken meat and environmental samples were reported (12, 49, 51). In the isolated *E. coli* and *Salmonella*, the *qnrA* gene was detected in 9.6% and 11.2% of *E. coli* and *Salmonella* spp. isolates from retail chicken meat, respectively. These findings differ from a similar previous study in South Korea, where they reported that none of the *E. coli* and *Salmonella* harbored the *qnrA* gene in retail chicken meat (49, 63). Furthermore, 21.6% of *E. coli* isolates from LBM sewage samples also harbored the *qnrA* gene, which differs from the study reported earlier in India (51). Moreover, the *qnrB* gene was identified in 2.2% of the *E. coli* and in 2.2% of *Salmonella* isolates from retail chicken meat, which is consistent with the previous report conducted in South Korea, where the *qnrB* gene was detected in 2.6% of *Salmonella* isolates from retail chicken meat (64), but in contrast with the report from Egypt, where *qnrB* was found in 33.3% of *E. coli* and 20% of *Salmonella* isolates (65). The prevalence of the *qnrS* gene seems to be overall higher than that of the other *qnr* genes in the current study, which could represent a significant resistance trait along the food chain. Plasmids harboring *qnr* genes may contribute to the horizontal spread of antibiotic resistance in poultry and in the environment. The high prevalence of *qnrA* and *qnrS* genes likely reflects the indiscriminate and widespread use of fluoroquinolones in veterinary practices and over-the-counter access. Clinically, these results are significant because PMQR genes, though conferring only low-level resistance, facilitate the emergence of higher-level fluoroquinolone resistance (66). These results highlight the need for cautious use of fluoroquinolones to prevent the spread of resistance.

In this study, the coexistence of two or more genes in the same strain was common in *E. coli* and *Salmonella* isolated from retail chicken meat and LBM sewage samples. The most frequent coexistence among *E. coli* and *Salmonella* isolates from both types of samples was *bla_TEM* and *qnrS*. The coexistence of different β-lactamase and PMQR genes within the same isolates has been reported by several investigators (51, 60, 64). It has been demonstrated that fluoroquinolone resistance can be mediated by co-transfer of the *qnr* determinant on β-lactamase, mainly ESBL-carrying plasmids (67). Co-transmission of β-lactamase and fluoroquinolone resistance is clinically important because co-selection of resistance by use of either drug may occur. This complicates treatment options and heightens the risk of therapeutic failure in clinical infections caused by *E. coli* (68).

The present study revealed several strong associations between AMR phenotypes, particularly between fluoroquinolones and β-lactam antibiotics, in *E. coli* and *Salmonella* isolated from retail chicken meat and LBM sewage. These associations are biologically plausible given that fluoroquinolones and β-lactam antibiotics share similar targets and resistance determinants, often residing on mobile genetic elements (68, 69). The exceptionally high odds ratios were observed between certain phenotype pairs (e.g., LEV/CIP; GAT/LEV; NA/LEV; CIP/GAT; NOR/OFX; OFX/LEV; NA/NOR; FEP/ATM; CAZ/FEP; CXM/FEP; ATM/CAZ; CL/CE; ATM/FEP; CT/PB; CRO/CFM; CXM/CL; NA/PEF; CFM/CL; and AM/SXT), indicating that resistance to one antimicrobial agent is highly predictive of resistance to one or more additional agents (7).

This study also described the association between AMR phenotypes and AMR genes in *E. coli* and *Salmonella* isolated from retail chicken meat and LBM sewage samples, revealing that not only the phenotypically resistant isolates carried the respective β-lactamase or PMQR genes but also the phenotypically non-resistant isolates of *E. coli* and *Salmonella* carried the resistance genes, and some isolates showed strong association between AMR phenotypes and genotypes in these two bacteria. Resistance genes are often associated with integrons or transposons, mobile DNA elements that facilitate their spread (70). The strong associations observed between phenotypes and ARGs support the idea that many ARGs are linked on such elements. Previous studies also reported high statistical associations between resistance genes, which were frequently confirmed by molecular evidence (71). However, the exact mechanisms of association are unknown in the current study. Certain phenotypes and genotypes showed particularly strong associations, suggesting that resistance was often attributable to a single gene, such as ceftazidime and *bla_CTX-M-1*.

However, neither phenotype nor genotype alone reliably predicted resistance. AMR mechanisms are complex, and the presence or lack of a specific gene does not always determine phenotype (7). Both resistant and non-resistant *E. coli* and *Salmonella* isolates carried β-lactamase or PMQR genes, though some showed strong phenotype–genotype associations. The mismatch between genotype and phenotype may reflect untested resistance genes or genes not expressed in particular isolates. Because ARGs can cluster on mobile elements (plasmids, transposons, and integrons), use of one antimicrobial may co-select for resistance to multiple drug classes (71, 72). Thus, even restricted antimicrobial use may maintain ARGs through co-selection (7).

Some ARGs, such as *bla_TEM*, showed no associations with any of the resistance phenotypes in both *E. coli* and *Salmonella* isolated from retail chicken meat and LBM sewage. This lack of correlation may be attributed to the high prevalence or ubiquity of this gene within the bacterial populations (73, 74). When an ARG is widespread across many isolates, including both resistant and susceptible isolates, it becomes difficult to establish a direct correlation between its presence and the observed resistance phenotype. This ubiquity can mask the gene’s functional impact, as factors like gene expression levels, regulatory mechanisms, or the presence of additional resistance determinants influence whether the gene actually confers a measurable resistance phenotype (69, 73). Consequently, the high prevalence of *bla_TEM* may lead to an apparent disconnect between genotype and phenotype, making associations less apparent or statistically insignificant.

The phylogenetic analysis of β-lactamase genes revealed that all study isolates were clearly grouped into BSBL (*bla_TEM* and *bla_SHV*) and ESBL (*bla_CTX-M-1* and *bla_CTX-M-2*) groups. The continuous exposure of bacterial strains to β-lactams has led to dynamic and massive production and mutation of β-lactamases. ESBLs are derived from point mutations in the *bla_TEM-1* and *bla_SHV-1* β-lactamase genes (75). The amino acid sequences of ESBLs often differ from those of the parent enzymes, usually TEM-1, TEM-2, and SHV-1, by only one, two, or three amino acids (5). However, we did not observe any nucleotide and amino acid substitutions among the sequences of the same family.

Nucleotide and amino acid substitutions of the PMQR genes were analyzed with reference sequences. No nucleotide and amino acid substitutions were detected among the sequences of the *qnrA* and *qnrS* groups. However, the results of sequence comparison of the *qnrB* gene revealed several amino acid substitutions at different positions (79 [S > A and V], 142 [I > M], 94 [A > S], 144 [A > T], 163 [T > S], 186 [I > V], and 198 [N > S]). Although these substitutions currently have no established regulatory roles and their functional significance remains unclear, changes occurring within critical domains, such as loop B or the structural core, may potentially affect the protective activity of the *qnr* protein (76, 77).

In this study, *qnrB* genes were found to be associated with different mobile genetic elements, including the orf1005 gene encoding a putative transposase in *qnrB1* (78), the ISCR1 element in *qnrB2* (78, 79), and *qnrB12* (80), highlighting their potential for horizontal dissemination. Phylogenetic analysis of PMQR genes showed clear clustering into three distinct groups corresponding to *qnrA*, *qnrB*, and *qnrS*. Following the initial identification of *qnrA*, additional plasmid-mediated qnr variants, including *qnrS* (81), *qnrB* (78), *qnrC* (82), and *qnrD* (83), have since been described.

In conclusion, this study revealed robust networks of associations among antimicrobial resistance phenotypes, resistance genes, and phenotype–genotype relationships, indicating strong co-selection of fluoroquinolone and β-lactam resistance traits under antimicrobial pressure. The detection of resistance genes in phenotypically susceptible isolates further suggests the presence of silent AMR reservoirs that may be expressed under selective conditions, complicating surveillance and control efforts. Importantly, the resistance association framework generated here provides a strong evidence base for informing antimicrobial policy and clinical decision-making in Bangladesh. Retail chicken meat and live bird market environments were identified as significant reservoirs of multidrug-resistant *E. coli* and *Salmonella* carrying diverse AMR determinants with potential for transmission to humans and the environment. These findings underscore the urgent need for strengthened antimicrobial stewardship, improved hygiene and waste management in live bird markets, and integrated One Health-based phenotypic and genotypic surveillance to effectively curb the spread of AMR within the poultry production system in Bangladesh.

## Supplementary Material

Reviewer comments

## Data Availability

The data sets generated and analyzed during the current study are available from the corresponding author on reasonable request.

## SUPPLEMENTAL MATERIAL

Table S1; Fig. S1 and S2 spectrum.01062-25-s0001.docx Table S1: List of antimicrobials. Fig. S1 and S2: Comparative alignment for nucleotide and amino acids of 16 *E. coli* strains.

OPEN PEER REVIEW reviewer-comments.pdf An accounting of the reviewer comments and feedback.

ASM does not own the copyrights to Supplemental Material that may be linked to, or accessed through, an article. The authors have granted ASM a non-exclusive, world-wide license to publish the Supplemental Material files. Please contact the corresponding author directly for reuse.
