# Supplementary material for: Association between phenotypes and genotypes of antimicrobial resistance in E. coli and Salmonella isolates from retail chicken meat and live bird market sewage in Bangladesh
Source: Microbiol Spectr. 2026 Jun 30;14(8):e01062-25. doi: 10.1128/spectrum.01062-25 (PMC13436203; doi:10.1128/spectrum.01062-25)
Supplement: Supplementary file 1 [file reviewer-comments.pdf]

AMERICAN SOCIETY FOR MICROBIOLOGY | Microbiology Spectrum

Association between phenotypes and genotypes of antimicrobial resistance in *E. coli* and *Salmonella* isolates from retail chicken meat and live bird market sewage in Bangladesh

Mst. Sonia Parvin, Sudipta Talukder, Sayra Tasnin Sharmy, Md. Mehedi Hasan, and Md. Taohidul Islam

Corresponding Author(s): Md. Taohidul Islam, Bangladesh Agricultural University

Review Timeline:

Submission Date:	April 7, 2025
Editorial Decision:	July 1, 2025
Revision Received:	September 4, 2025
Editorial Decision:	December 19, 2025
Revision Received:	February 10, 2026
Editorial Decision:	February 11, 2026
Revision Received:	February 13, 2026
Accepted:	February 22, 2026

Editor: Salina Parveen

Reviewer(s): Disclosure of reviewer identity is with reference to reviewer comments included in decision letter(s). The following individuals involved in review of your submission have agreed to reveal their identity: Zajeba Tabashum (Reviewer #1); Chanelle Acheamfour (Reviewer #2)

Transaction Report:

(Note: With the exception of the correction of typographical or spelling errors that could be a source of ambiguity, letters and reports are not edited. The original formatting of letters and referee reports may not be reflected in this compilation.)

DOI: https://doi.org/10.1128/spectrum.01062-25

Re: Spectrum01062-25 (Association between phenotypes and genotypes of antimicrobial resistance in *E. coli* and *Salmonella* isolates from retail chicken meat and live bird market sewage in Bangladesh)

Dear Prof. Md. Taohidul Islam:

Thank you for the privilege of reviewing your work. Below you will find my comments, instructions from the Spectrum editorial office, and the reviewer comments.

Reviewers has concerns about results, discussion and significance of the study and grammatical errors.

I suggest the authors have the manuscript proofread and edited/revised by a native English speaker or a professional editing service.

Please return the manuscript within 60 days; if you cannot complete the modification within this time period, please contact me. If you do not wish to modify the manuscript and prefer to submit it to another journal, notify me immediately so that the manuscript may be formally withdrawn from consideration by Spectrum.

Revision Guidelines
To submit your modified manuscript, log into the submission site at https://spectrum.msubmit.net/cgi-bin/main.plex. Go to Author Tasks and click the appropriate manuscript title to begin. The information you entered when you first submitted the paper will be displayed; update this as necessary. Note the following requirements:

• Upload point-by-point responses to the issues raised by the reviewers in a file named "Response to Reviewers," NOT in your cover letter.
• Upload a compare copy of the manuscript (without figures) as a "Marked-Up Manuscript" file.
• Upload a clean .DOC/.DOCX version of the revised manuscript and remove the previous version.
• Each figure must be uploaded as a separate, editable, high-resolution file (TIFF or EPS preferred), and any multipanel figures must be assembled into one file.
• Any supplemental material intended for posting by ASM should be uploaded with their legends separate from the main manuscript. You can combine all supplemental material into one file (preferred) or split it into a maximum of 10 files with all associated legends included.

For complete guidelines on revision requirements, see our Submission and Review Process webpage. Submission of a paper that does not conform to guidelines may delay acceptance of your manuscript.

Data availability: ASM policy requires that data be available to the public upon online posting of the article, so please verify all links to sequence records, if present, and make sure that each number retrieves the full record of the data. If a new accession number is not linked or a link is broken, provide Spectrum production staff with the correct URL for the record. If the accession numbers for new data are not publicly accessible before the expected online posting of the article, publication may be delayed; please contact production staff (Spectrum@asmusa.org) immediately with the expected release date.

Publication Fees: For information on publication fees and which article types are subject to charges, visit our website. If your manuscript is accepted for publication and any fees apply, you will be contacted separately about payment during the production process; please follow the instructions in that e-mail. Arrangements for payment must be made before your article is published.

ASM Membership: Corresponding authors may join or renew ASM membership to obtain discounts on publication fees. Need to upgrade your membership level? Please contact Customer Service at Service@asmusa.org.

The ASM Journals program strives for constant improvement in our submission and publication process. Please tell us how we can improve your experience by taking this quick Author Survey.

Thank you for submitting your paper to Spectrum.

Sincerely,
Salina Parveen
Editor
Microbiology Spectrum

Reviewer #1 (Comments for the Author):

Major concern:
• Figures are referenced multiple times, a brief verbal summary of the figures would help the narrative. Mot just mere numbers.
• Important comparisons between E. coli and Salmonella could be made clearer-especially regarding which drugs showed relatively unique resistance patterns.
• Specify the clinical significance of each gene e.g., blaTEM confers resistance to broad-spectrum β-lactams.
• Adding a sentence on why coexistence matters for public health would strengthen the implications.
• Because some ORs are extremely high, briefly interpret what this means biologically
• Clarify why some ARGs like blaTEM showed no associations-could this be due to high prevalence (ubiquity) or lack of functional interplay?
• Some negative associations (e.g., phenotypically susceptible isolates carrying resistance genes) are counterintuitive and deserve more explanation. Add a brief comment on possible reasons (e.g., gene silencing, low expression levels, or compensatory mutations).
• Clarify the significance of the observed nucleotide substitutions, especially since no amino acid changes were noted-do these substitutions have known regulatory roles?
• When discussing associations (e.g., phenotype-genotype), mention the specific statistical tests used to determine significance.
• Explain "strong associations" what threshold or statistical method defines them?

Minor concern:
• Consider defining abbreviations the first time they appear and only once (e.g., ARGs).
• Be more concise in places to avoid repetition (e.g., you mention the same role of E. coli and Salmonella in multiple ways).
• Replace passive voice with active where possible.
• Clarify sequencing targets and numbers more cleanly.
• Standardize units (e.g., μg), gene formatting (e.g., bla_TEM), and organism names (E. coli, Salmonella).
• Some sentences are overly long and dense with numerical data, which can reduce readability.

Reviewer #2 (Comments for the Author):

The manuscript by Parvin et al., investigated antimicrobial resistance genes in E. coli and Salmonella isolated from retail chicken meat and live bird market sewage in Bangladesh. This manuscript suffers from numerous grammatical errors, incorrect word usage, punctuation problems and inconsistences, and run-on sentences (too numerous to be listed in the comments). I suggest the authors have the manuscript proofread and edited by a native English speaker or a professional editing service.

There were concerns that the English language usage in the manuscript might make it difficult to properly evaluate the science. The ASM Journals webpage provides links to various language editing services (https://journals.asm.org/writing-your-paper#language-editing-services). You may consider using these services when revising your manuscript. The use of these services will have no direct bearing on the editorial decision. ASM has no affiliation with these companies.

Major concern:

- Figures are referenced multiple times, a brief verbal summary of the figures would help the narrative. Mot just mere numbers.
- Important comparisons between *E. coli* and *Salmonella* could be made clearer—especially regarding which drugs showed relatively unique resistance patterns.
- Specify the clinical significance of each gene e.g., blaTEM confers resistance to broad-spectrum β-lactams.
- Adding a sentence on why coexistence matters for public health would strengthen the implications.
- Because some ORs are extremely high, briefly interpret what this means biologically
- Clarify why some ARGs like *blaTEM* showed no associations—could this be due to high prevalence (ubiquity) or lack of functional interplay?
- Some negative associations (e.g., phenotypically susceptible isolates carrying resistance genes) are counterintuitive and deserve more explanation. Add a brief comment on possible reasons (e.g., gene silencing, low expression levels, or compensatory mutations).
- Clarify the significance of the observed nucleotide substitutions, especially since no amino acid changes were noted—do these substitutions have known regulatory roles?
- When discussing associations (e.g., phenotype-genotype), mention the specific statistical tests used to determine significance.
- Explain "strong associations" what threshold or statistical method defines them?

Minor concern:

- Consider defining abbreviations the first time they appear and only once (e.g., ARGs).
- Be more concise in places to avoid repetition (e.g., you mention the same role of *E. coli* and *Salmonella* in multiple ways).
- Replace passive voice with active where possible.
- Clarify sequencing targets and numbers more cleanly.
- Standardize units (e.g., µg), gene formatting (e.g., *bla_TEM*), and organism names (*E. coli*, *Salmonella*).
- Some sentences are overly long and dense with numerical data, which can reduce readability.

Response Note

Reviewer #1 (Comments for the Author):

Major concern:

Figures are referenced multiple times; a brief verbal summary of the figures would help the narrative. Not just mere numbers.

Response: We have revised throughout the texts.

Important comparisons between *E. coli* and *Salmonella* could be made clearer—especially regarding which drugs showed relatively unique resistance patterns.

Response: We have clarified the comparisons between E. coli and Salmonella by highlighting both the shared and distinct resistance patterns (Line 270-306, 514-558).

Specify the clinical significance of each gene e.g., blaTEM confers resistance to broad-spectrum β-lactams.

Response: We have revised the manuscript to include a brief description of the clinical relevance of each detected gene in the introduction (Line 100-111).

Adding a sentence on why coexistence matters for public health would strengthen the implications.

Response: Added (Line 595-598).

Because some ORs are extremely high, briefly interpret what this means biologically

Response: Interpretation added (Line 599-612).

Clarify why some ARGs like *blaTEM* showed no associations—could this be due to high prevalence (ubiquity) or lack of functional interplay?

Response: We discussed to clarify to reflect these possibilities (Line 643-653).

Some negative associations (e.g., phenotypically susceptible isolates carrying resistance genes) are counterintuitive and deserve more explanation. Add a brief comment on possible reasons (e.g., gene silencing, low expression levels, or compensatory mutations).

Response: We have added a brief explanation discussing possible underlying reasons. Relevant references supporting these mechanisms have also been included to provide additional context. (Line 634-641).

Clarify the significance of the observed nucleotide substitutions, especially since no amino acid changes were noted—do these substitutions have known regulatory roles?

Response: Revised (Line 686-690).

When discussing associations (e.g., phenotype-genotype), mention the specific statistical tests used to determine significance.

Response: Revised (Line 335-342).

Explain "strong associations" what threshold or statistical method defines them?

Response: The definition of strong associations has been added to the statistical analysis section of the materials and methods (Line 248-251).

Minor concern:

Consider defining abbreviations the first time they appear and only once (e.g., ARGs).

Response: We have now defined all abbreviations at their first appearance in the manuscript and ensured they are only defined once.

Be more concise in places to avoid repetition (e.g., you mention the same role of *E. coli* and *Salmonella* in multiple ways).

Response: We have revised the manuscript to avoid repetitive statements regarding the roles of *E. coli* and *Salmonella*. The relevant sections have been consolidated to present the information more concisely without loss of clarity.

Replace passive voice with active where possible.

Response: Revised throughout the texts.

Clarify sequencing targets and numbers more cleanly.

Response: Incorporated (Fig. 4-7, Line 999-1010)

Standardize units (e.g., µg), gene formatting (e.g., *bla_TEM*), and organism names (*E. coli*, *Salmonella*).

Response: Revised.

Some sentences are overly long and dense with numerical data, which can reduce readability.

Response: Revised throughout the texts.

Reviewer #2 (Comments for the Author):

The manuscript by Parvin et al., investigated antimicrobial resistance genes in E. coli and Salmonella isolated from retail chicken meat and live bird market sewage in Bangladesh. This manuscript suffers from numerous grammatical errors, incorrect word usage, punctuation problems and inconsistences, and run-on sentences (too numerous to be listed in the comments). I suggest the authors have the manuscript proofread and edited by a native English speaker or a professional editing service.

Response: Thank you very much for your concern regarding the language quality of our manuscript. We acknowledge the presence of grammatical errors, inconsistent punctuation, and incorrect word usage as noted. In response to this comment, we have thoroughly revised the entire manuscript to address these issues. The revised version has been carefully proofread and edited by a native speaker to ensure clarity, consistency, and correctness in language usage.

Re: Spectrum01062-25R1 (Association between phenotypes and genotypes of antimicrobial resistance in *E. coli* and *Salmonella* isolates from retail chicken meat and live bird market sewage in Bangladesh)

Dear Prof. Md. Taohidul Islam:

Thank you for the privilege of reviewing your work. Below you will find my comments, instructions from the Spectrum editorial office, and the reviewer comments.

The revised version reads better than the previous one. However, both reviewers have raised concerns regarding the clarity and flow of the manuscript. In addition, please address a few minor comments from Reviewer 1.

Please return the manuscript within 60 days; if you cannot complete the modification within this time period, please contact me. If you do not wish to modify the manuscript and prefer to submit it to another journal, notify me immediately so that the manuscript may be formally withdrawn from consideration by Spectrum.

Revision Guidelines
To submit your modified manuscript, log into the submission site at https://spectrum.msubmit.net/cgi-bin/main.plex. Go to Author Tasks and click the appropriate manuscript title to begin. The information you entered when you first submitted the paper will be displayed; update this as necessary. Note the following requirements:

• Upload point-by-point responses to the issues raised by the reviewers in a file named "Response to Reviewers," NOT in your cover letter.
• Upload a compare copy of the manuscript (without figures) as a "Marked-Up Manuscript" file.
• Upload a clean .DOC/.DOCX version of the revised manuscript and remove the previous version.
• Each figure must be uploaded as a separate, editable, high-resolution file (TIFF or EPS preferred), and any multipanel figures must be assembled into one file.
• Any supplemental material intended for posting by ASM should be uploaded with their legends separate from the main manuscript. You can combine all supplemental material into one file (preferred) or split it into a maximum of 10 files with all associated legends included.

For complete guidelines on revision requirements, see our Submission and Review Process webpage. Submission of a paper that does not conform to guidelines may delay acceptance of your manuscript.

Data availability: ASM policy requires that data be available to the public upon online posting of the article, so please verify all links to sequence records, if present, and make sure that each number retrieves the full record of the data. If a new accession number is not linked or a link is broken, provide Spectrum production staff with the correct URL for the record. If the accession numbers for new data are not publicly accessible before the expected online posting of the article, publication may be delayed; please contact production staff (Spectrum@asmusa.org) immediately with the expected release date.

Publication Fees: For information on publication fees and which article types are subject to charges, visit our website. If your manuscript is accepted for publication and any fees apply, you will be contacted separately about payment during the production process; please follow the instructions in that e-mail. Arrangements for payment must be made before your article is published.

ASM Membership: Corresponding authors may join or renew ASM membership to obtain discounts on publication fees. Need to upgrade your membership level? Please contact Customer Service at Service@asmusa.org.

The ASM Journals program strives for constant improvement in our submission and publication process. Please tell us how we can improve your experience by taking this quick Author Survey.

Thank you for submitting your paper to Spectrum.

Sincerely,
Salina Parveen
Editor
Microbiology Spectrum

Reviewer #1 (Comments for the Author):

The manuscript has improved substantially since the initial submission. However, further linguistic refinement is required to improve readability, clarity, and flow, particularly in some sections where sentence structure and wording may obscure the intended meaning.

The manuscript has improved substantially since the initial submission. However, further linguistic modification is required to improve readability, clarity, and flow, particularly in some sections where sentence structure and wording may hinder the intended meaning.

Reviewer #1 (Comments for the Author):

The manuscript has improved substantially since the initial submission. However, further linguistic refinement is required to improve readability, clarity, and flow, particularly in some sections where sentence structure and wording may obscure the intended meaning.

Response: We sincerely thank the reviewer for the positive evaluation and constructive comment regarding the overall improvement of the manuscript.

We have carefully revised the entire manuscript to further enhance readability, clarity, and logical flow. Particular attention was given to sections where sentence structure or wording could potentially obscure the intended meaning. We refined phrasing, simplified complex sentences, improved transitions between ideas, and ensured consistency in terminology throughout the text. These revisions were made without altering the scientific content or interpretation of the results.

Re: Spectrum01062-25R2 (Association between phenotypes and genotypes of antimicrobial resistance in *E. coli* and *Salmonella* isolates from retail chicken meat and live bird market sewage in Bangladesh)

Dear Prof. Md. Taohidul Islam:

Thank you for the privilege of reviewing your work. Below you will find my comments, instructions from the Spectrum editorial office, and the reviewer comments.

Thank you so much for addressing the reviewer # 1 comments.
Please address following comments of the reviewer 2.

"The authors have made substantial improvements to the manuscript, and the overall quality has notably increased. However, a few minor revisions are still required to enhance clarity, precision, and consistency throughout the text.
Line 67: The term "researches" is not appropriate in this context. Please replace it with "studies" or "research work."
Lines 136-138: The sentence structure is cumbersome and repetitive. Consider revising to the following for clarity and conciseness:
"Between 2018 and 2020, 320 retail chicken meat samples and 64 sewage samples were collected from live bird markets (LBMs) located in 32 upazilas across 16 districts in 8 divisions of Bangladesh."
Line 140: The phrase "tiny, fine pieces" is redundant. Retain only one descriptor ("tiny pieces" or "fine pieces").
Lines 140-141: The authors need to specify what contained 225 mL of buffered peptone water
Line 142: The transitional word "Besides" is not appropriate. Replace it with "Additionally" or "Furthermore," depending on the intended meaning.
Lines 170-185: The long list of 31 antimicrobial agents would be clearer if presented in table format, with columns for antimicrobial class, agent, abbreviation, and disc concentration.
Line 248: The abbreviation "OR" should be written out in full at first use as "Odds Ratio (OR)."
Line 577: Insert the word "and" between E. coli and Salmonella for grammatical correctness.
Line 579: The word "detected" is misspelled. In addition, please clarify whether these were E. coli and Salmonella isolates.
Line 580: A space should be inserted between "gene" and "appeared."
Line 591: Replace "both species" with "both genera," as the reference is to E. coli and Salmonella.
Line 672: Add a space between "reported" and "in."
Line 716: The word "sources" should be changed to the singular form "source."

Please return the manuscript within 60 days; if you cannot complete the modification within this time period, please contact me. If you do not wish to modify the manuscript and prefer to submit it to another journal, notify me immediately so that the manuscript may be formally withdrawn from consideration by Spectrum.

Revision Guidelines
To submit your modified manuscript, log into the submission site at https://spectrum.msubmit.net/cgi-bin/main.plex. Go to Author Tasks and click the appropriate manuscript title to begin. The information you entered when you first submitted the paper will be displayed; update this as necessary. Note the following requirements:

- Upload point-by-point responses to the issues raised by the reviewers in a file named "Response to Reviewers," NOT in your cover letter.
- Upload a compare copy of the manuscript (without figures) as a "Marked-Up Manuscript" file.
- Upload a clean .DOC/.DOCX version of the revised manuscript and remove the previous version.
- Each figure must be uploaded as a separate, editable, high-resolution file (TIFF or EPS preferred), and any multipanel figures must be assembled into one file.
- Any supplemental material intended for posting by ASM should be uploaded with their legends separate from the main manuscript. You can combine all supplemental material into one file (preferred) or split it into a maximum of 10 files with all associated legends included.

For complete guidelines on revision requirements, see our Submission and Review Process webpage. Submission of a paper that does not conform to guidelines may delay acceptance of your manuscript.

Data availability: ASM policy requires that data be available to the public upon online posting of the article, so please verify all links to sequence records, if present, and make sure that each number retrieves the full record of the data. If a new accession number is not linked or a link is broken, provide Spectrum production staff with the correct URL for the record. If the accession numbers for new data are not publicly accessible before the expected online posting of the article, publication may be delayed; please contact production staff (Spectrum@asmusa.org) immediately with the expected release date.

Publication Fees: For information on publication fees and which article types are subject to charges, visit ourwebsite. If your manuscript is accepted for publication and any fees apply, you will be contacted separately about payment during the production

process; please follow the instructions in that e-mail. Arrangements for payment must be made before your article is published.

ASM Membership: Corresponding authors may join or renew ASM membership to obtain discounts on publication fees. Need to upgrade your membership level? Please contact Customer Service at Service@asmusa.org.

The ASM Journals program strives for constant improvement in our submission and publication process. Please tell us how we can improve your experience by taking this quick Author Survey.

Thank you for submitting your paper to Spectrum.

Sincerely,
Salina Parveen
Editor
Microbiology Spectrum

Reviewer #2 (Comments for the author):

Line 67: The term "researches" is not appropriate in this context. Please replace it with "studies" or “research work”.

Response: Replaced.

Lines 136-138: The sentence structure is cumbersome and repetitive. Consider revising to the following for clarity and conciseness:

Between 2018 and 2020, 320 retail chicken meat samples and 64 sewage samples were collected from live bird markets (LBMs) located in 32 upazilas across 16 districts in 8 divisions of Bangladesh.

Response: Revised.

Line 140: The phrase "tiny, fine pieces" is redundant. Retain only one descriptor ("tiny pieces" or "fine pieces").

Response: Revised.

Lines 140-141: The authors need to specify what contained 225 mL of buffered peptone water

Response: We have revised as ‘......25 g chicken meat sample was chopped into fine pieces, aseptically transferred into a sterile glass beaker containing 225 mL of buffered peptone water (BPW).......’.

Line 142: The transitional word "Besides" is not appropriate. Replace it with "Additionally" or "Furthermore," depending on the intended meaning.

Response: Replaced with ‘Furthermore’.

Lines 170-185: The long list of 31 antimicrobial agents would be clearer if presented in table format, with columns for antimicrobial class, agent, abbreviation, and disc concentration.

Response: We have presented the list of antimicrobials in a table format.

Line 248: The abbreviation "OR" should be written out in full at first use as "Odds Ratio (OR)."

Response: Revised.

Line 577: Insert the word "and" between E. coli and Salmonella for grammatical correctness.

Response: Corrected.

Line 579: The word "detected" is misspelled. In addition, please clarify whether these were E. coli and Salmonella isolates.

Response: Revised. The clarification has been incorporated into the revised manuscript.

Line 580: A space should be inserted between "gene" and "appeared."

Line 591: Replace "both species" with "both genera," as the reference is to E. coli and Salmonella.

Line 672: Add a space between "reported" and "in."

Line 716: The word "sources" should be changed to the singular form "source."

Response (Lines 580, 591, 672, and 716): We thank the reviewer for these careful observations. The issues mentioned were present in the first revised version but have already been removed in the second revised manuscript. Therefore, no further changes were required in the current revision.

Re: Spectrum01062-25R3 (Association between phenotypes and genotypes of antimicrobial resistance in *E. coli* and *Salmonella* isolates from retail chicken meat and live bird market sewage in Bangladesh)

Dear Prof. Md. Taohidul Islam:

Your manuscript has been accepted, and I am forwarding it to the ASM production staff for publication. Your paper will first be checked to make sure all elements meet the technical requirements. ASM staff will contact you if anything needs to be revised before copyediting and production can begin. Otherwise, you will be notified when your proofs are ready to be viewed.

Data Availability: ASM policy requires that data be available to the public upon online posting of the article, so please verify all links to sequence records, if present, and make sure that each number retrieves the full record of the data. If a new accession number is not linked or a link is broken, provide production staff with the correct URL for the record. If the accession numbers for new data are not publicly accessible before the expected online posting of the article, publication may be delayed; please contact ASM production staff immediately with the expected release date.

Publication Fees: For information on publication fees and which article types have charges, please visit our website. We have partnered with Copyright Clearance Center (CCC) to collect author charges. If fees apply to your paper, you will receive a message from no-reply@copyright.com with further instructions. For questions related to paying charges through RightsLink, please contact CCC at ASM_Support@copyright.com or toll free at +1-877-622-5543. CCC makes every attempt to respond to all emails within 24 hours.

ASM Membership: Corresponding authors may join or renew ASM membership to obtain discounts on publication fees. Need to upgrade your membership level? Please contact Customer Service at Service@asmusa.org.

Embargo Policy: A press release may be issued as soon as the manuscript is posted on the Spectrum Latest Articles webpage. The corresponding author will receive an email with the subject line "ASM Journals Author Services Notification" when the article is available online.

The ASM Journals program strives for constant improvement in our submission and publication process. Please tell us how we can improve your experience by taking this quick Author Survey.

Thank you for submitting your paper to Spectrum.

Sincerely,
Salina Parveen
Editor
Microbiology Spectrum
