# Supplementary material for: Association between phenotypes and genotypes of antimicrobial resistance in E. coli and Salmonella isolates from retail chicken meat and live bird market sewage in Bangladesh
Source: Microbiol Spectr. 2026 Jun 30;14(8):e01062-25. doi: 10.1128/spectrum.01062-25 (PMC13436203; doi:10.1128/spectrum.01062-25)
Supplement: Supplementary file 2 [file spectrum.01062-25-s0001.docx]

**Supplementary Table S1.** List of antimicrobials used for antimicrobial susceptibility testing

| **SN** | **Antimicrobial classes** | **Antimicrobial agents** | **Abbreviation** | **Disk concentration (µg)** |
| --- | --- | --- | --- | --- |
| 1. | Fluoroquinolones | Nalidixic acid | NA | 30 |
|  |  | Ciprofloxacin | CIP | 5 |
|  |  | Levofloxacin | LEV | 5 |
|  |  | Norfloxacin | NOR | 10 |
|  |  | Ofloxacin | OFX | 5 |
|  |  | Gatifloxacin | GAT | 5 |
|  |  | Pefloxacin | PEF | 5 |
| 2. | Non-extended spectrum cephalosporins | | | |
|  | First-generation cephalosporins | Cephalexin | CL | 30 |
|  |  | Cephradine | CE | 30 |
|  | Second-generation cephalosporins | Cefuroxime | CXM | 30 |
|  |  | Cefaclor | CEC | 30 |
| 3. | Extended-spectrum cephalosporins | | | |
|  | Third-generation cephalosporins | Cefotaxime | CTX | 30 |
|  |  | Ceftriaxone | CRO | 30 |
|  |  | Ceftazidime | CAZ | 30 |
|  |  | Cefixime | CFM | 5 |
|  | Fourth-generation cephalosporins | Cefepime | FEP | 30 |
| 4. | Cephamycins | Cefoxitin | FOX | 30 |
| 5. | Penicillins | Ampicillin | AM | 10 |
| 6. | Penicillins + β-lactamase inhibitors | Amoxicillin-clavulanic acid | AMC | 30 |
| 7. | Antipseudomonal penicillins + β-lactamase inhibitors | Pipercillin-tazobactam | TPZ | 110 |
| 8. | Carbapenems | Imipenem | IPM | 10 |
|  |  | Meropenem | MEM | 10 |
| 9. | Monobactams | Aztreonam | ATM | 30 |
| 10. | Aminoglycosides | Gentamicin | CN | 10 |
|  |  | Amikacin | AK | 30 |
| 11. | Tetracyclines | Doxycycline | DO | 10 |
| 12. | Folate pathway inhibitors | Trimethoprim-sulfamethoxazole | SXT | 25 |
| 13. | Glycylcyclines | Tigecycline | TGC | 15 |
| 14. | Phenicols | Chloramphenicol | C | 30 |
| 15. | Polymyxins | Colistin | CT | - |
|  |  | Polymyxin B | PB | - |

(-), Minimum inhibitory concentration was determined for these two antibiotics.

**Supplementary Fig. S1.** Comparative alignment for nucleotide comparison of 16 *E. coli* strains. Note: The nucleotide of sequence K-12: MG1655 (GenBank Accession no: NC_000913.3) on the top is used as a reference sequence. Identity to the top sequence is marked by dot (.) and difference is presented by abbreviated letter of nucleotide itself.

**Supplementary Fig. S2.** Comparative alignment for amino acid comparison of 16 *E. coli* strains. Note: The amino acid of sequence K-12: MG1655 (GenBank Accession no: NC_000913.3) on the top is used as a reference sequence. Identity to the top sequence is marked by dot (.).

**:** 131

**:** 131

**:** 131

**:** 124

**:** 131

**:** 131

**:** 123

**:** 131

**:** 131

**:** 123

**:** 131

**:** 127

**:** 131

**:** 124

**:** 131

**:** 122

**:** 131

**:**

**:**

**:**

**:**

**:**

**:**

**:**

**:**

**:**

**:**

**:**

**:**

**:**

**:**

**:**

**:**

**:**

**:** 131

**:** 131

**:** 131

**:** 124

**:** 131

**:** 131

**:** 123

**:** 131

**:** 131

**:** 123

**:** 131

**:** 127

**:** 131

**:** 124

**:** 131

**:** 122

**:** 131

**:**

**:**

**:**

**:**

**:**

**:**

**:**

**:**

**:**

**:**

**:**

**:**

**:**

**:**

**:**

**:**

**:**
